# Supplementary material for: Description of a new species of cryptic snubnose darter (Percidae: Etheostomatinae) endemic to north-central Mississippi
Source: PeerJ. 2020 Aug 31;8:e9807. doi: 10.7717/peerj.9807 (PMC7469936; doi:10.7717/peerj.9807)
Supplement: Supplemental Information 1 [file peerj-08-9807-s001.pdf]

Description of a New Species of Cryptic Snubnose Darter (Percidae: Etheostomatinae) Endemic to North-Central Mississippi

Ken A. Sterling[1*] and Melvin L. Warren, Jr[1].

[1]USDA Forest Service, Southern Research Station, Stream Ecology Laboratory, 1000 Front Street, Oxford, MS 38655

[*]*corresponding author*: kenneth.a.sterling@usda.gov

Supplemental tables and figures

Table S1. Estimated stream gradients and watershed area for Yazoo Darters in the Yocona (Y.R.) and Little Tallahatchie (L.T.R.) river drainages. Data is for all streams yielding Yazoo Darters but not including collections from the mainstem L.T.R. or Otoucalofa Creek (see Sterling et al., 2013); U.T. = unnamed tributary, Cr. = creek

Drainage	Stream	Lat.	Long.	Area (km²)	Gradient
Y.R.	U.T. Otoucalofa Cr. 2	34.141	-89.589	3.43	0.920
Y.R.	Mill Cr.	34.166	-89.52	5.6	0.933
Y.R.	Moore Cr.	34.156	-89.548	8.1	0.958
Y.R.	Shippy Cr.	34.153	-89.433	8.7	0.681
Y.R.	U.T. Otoucalofa Cr. 1	34.125	-89.61	10.1	0.874
Y.R.	Gordon Branch	34.14	-89.549	12.3	1.139
Y.R.	Morris Cr.	34.283	-89.544	13.5	0.555
Y.R.	Spring Cr.	34.153	-89.529	13.9	0.876
Y.R.	Smith Cr. B	34.138	-89.474	13.9	0.587
Y.R.	Smith Cr. A	34.168	-89.438	17.2	0.802
Y.R.	Johnston Cr.	34.123	-89.641	24.3	0.472
Y.R.	Taylor Cr.	34.296	-89.588	29.4	0.597
Y.R.	Splinter Cr.	34.235	-89.635	37.4	0.480
Y.R.	Pumpkin Cr.	34.327	-89.397	39.5	0.511
Y.R.	Yellow Leaf Cr.	34.375	-89.421	84.4	0.562
Y.R.	Tarvers Cr.	34.251	-89.489	26.1	0.730
Y.R.	U.T. Yocona River	34.273	-89.412	3.97	1.138
Y.R.	Kettle Cr.	34.3	-89.33	37.2	0.442
Y.R.	Long Branch	34.099	-89.803	37	0.482
L.T.R.	U.T. Tippah River 3	34.661	-89.288	4.43	1.306
L.T.R.	U.T. Tippah River1	34.691	-89.29	12.7	0.820
L.T.R.	U.T. Tippah River 2	34.709	-89.256	12.8	0.979
L.T.R.	Sorghum Cr.	34.707	-89.071	16	0.728
L.T.R.	Gray Cr.	34.719	-89.086	17.8	0.482
L.T.R.	Rhoden Cr.	34.756	-89.169	22	0.682
L.T.R.	Bagley Cr.	34.503	-89.413	27.5	0.634
L.T.R.	Fice Cr.	34.438	-89.253	28.9	0.414
L.T.R.	Graham Mill Cr.	34.503	-89.491	31.2	0.449
L.T.R.	Wagner Cr.	34.768	-89.229	32.9	0.462
L.T.R.	Mitchell Cr.	34.483	-89.203	34.4	0.349
L.T.R.	Yellow Rabbit Cr.	34.774	-89.145	41.9	0.452
L.T.R.	Chilli Cr.	34.682	-89.173	48.8	0.341

L.T.R.	Shelby Cr.	34.751	-89.102	49.6	0.230
L.T.R.	Oak Chewalla Cr.	34.582	-89.511	56.8	0.615
L.T.R.	Lee Cr.	34.513	-89.49	58.2	0.433
L.T.R.	Little Spring Cr.	34.642	-89.464	58.6	0.389
L.T.R.	Mill Cr.	34.525	-89.25	60.3	0.374
L.T.R.	Puskus Cr.	34.411	-89.375	66.4	0.325
L.T.R.	Bowling Branch	34.769	-88.997	66.4	0.482
L.T.R.	Hurricane Cr.	34.425	-89.496	76.1	0.399
L.T.R.	Blackwater Cr.	34.569	-89.61	82.9	0.546
L.T.R.	Cypress Cr.	34.394	-89.287	84.6	0.341
L.T.R.	Hotopha Cr. (Deer Cr.)	34.316	-89.785	89	0.345
L.T.R.	Big Snow Cr.	34.773	-89.252	97.7	0.348
L.T.R.	Chewalla Cr.	34.673	-89.332	118.6	0.340
L.T.R.	Big Spring Cr.	34.634	-89.397	119.7	0.311

Table S2. Diversity of clade Adonia snubnose darters showing described species, Evolutionary Significant Units (ESU), and undescribed but recognized forms; superscripts indicate possible cryptic diversity and citations, [1] = Storey (2003), Gabel (2007); [2] = Kozal et al. (2017); [3] = Boschung and Mayden (2004); [4] = Brogdon et al. (2003); colors correspond to unranked clades from Near et al. (2011).

	Species	Common Name
1	*Etheostoma scotti*	Cherokee Darter (upper ESU)
2	*Etheostoma scotti*	Cherokee Darter (middle ESU)
3	*Etheostoma scotti*	Cherokee Darter (lower ESU)
4	*Etheostoma coosae*[1]	Coosa Darter
5	*Etheostoma duryi*	Black Darter
6	*Etheostoma flavum*	Saffron Darter
7	*Etheostoma etnieri*	Cherry Darter
8	*Etheostoma raneyi*	Yazoo Darter
9	*Etheostoma faulkneri*	Yoknapatawpha Darter
10	*Etheostoma zonistium*[2]	Bandfin Darter
11	*Etheostoma cyanoprosopum*	Blueface Darter
12	*Etheostoma cf. zonistium*	Bandfin Darter: Hatchie River
13	*Etheostoma cervus*	Chickasaw Darter
14	*Etheostoma pyrrhogaster*	Firebelly Darter
15	*Etheostoma brevirostrum*	Holiday Darter: Shoal Creek
16	*Etheostoma cf. brevirostrum 1*	Holiday Darter Amicalola River
17	*Etheostoma cf. brevirostrum 2*	Holiday Darter: Conasauga River
18	*Etheostoma cf. brevirostrum 3*	Holiday Darter: Coosawattee River
19	*Etheostoma cf. brevirostrum 4*	Holiday Darter: Etowah River
20	*Etheostoma ramseyi*[3]	Alabama Darter
21	*Etheostoma tallapoosae*[4]	Tallapoosa Darter
22	*Etheostoma colorosum*	Coastal Darter
23	*Etheostoma lachneri*[3]	Tombigbee Darter
24	*Etheostoma bellator*	Warrior Darter
25	*Etheostoma cf. bellator 1*	Warrior Darter: Sipsey Fork
26	*Etheostoma cf. bellator 2*	Warrior Darter: Locust Fork
27	*Etheostoma chermocki*	Vermilion Darter

Table S3. Compilation of all known sites of occurrence for Yoknapatawpha Darters in the Yocona River (Y.R.) and Otoucalofa Creek (O.C.) watersheds; many sites have been sampled through time and the table does not reflect a complete compilation of all known sample records; Smith Creek A and Smith Creek B are different tributaries to Otoucalofa Creek; summer 1999 and summer 2012 indicates multiple samples over the summer; Cr. = creek, U.T. = unnamed tributary, SRS = USDA Forest Service, Southern Research Station (Oxford, MS).

Watershed	Stream	Lat.	Long.	Date	Source
O.C.	Gordon Branch	34.14	-89.549	6/15/1989	Suttkus et al., 1994
O.C.	Johnson Cr.	34.123	-89.641	6/19/2009	Sterling et al., 2013
O.C.	Mill Cr.	34.166	-89.52	6/18/2009	Sterling et al., 2013
O.C.	Moore Cr.	34.156	-89.548	6/18/2009	Sterling et al., 2013
O.C.	Otoucalofa Cr.	34.133	-89.412	7/8/1985	Ross et al., 2001
O.C.	Otoucalofa Cr.	34.162	-89.512	5/14/1986	Ross et al., 2001
O.C.	Shippy Cr.	34.153	-89.433	7/10/1985	Ross et al., 2001
O.C.	Smith Cr. A	34.168	-89.438	10/10/1985	Ross et al., 2001
O.C.	Smith Cr. A	34.171	-89.439	10/10/1985	Ross et al., 2001
O.C.	Smith Cr. B	34.138	-89.474	7/23/1985	Ross et al., 2001
O.C.	Spring Cr.	34.153	-89.529	6/30/2009	Sterling et al., 2013
O.C.	U.T. Otoucalofa Cr. 2	34.141	-89.589	6/14/1989	Suttkus et al., 1994
O.C.	U.T. Otoucalofa Cr. 1	34.125	-89.61	6/19/2009	Sterling et al., 2013
Y.R.	Kettle Cr.	34.3	-89.33	summer 1999	Adams & Warren 2005
Y.R.	Long Branch	34.099	-89.803	9/6/2015	Hubbell et al., 2020
Y.R.	Morris Cr.	34.3	-89.549	3/24/1993	Johnston & Haag 1996
Y.R.	Morris Cr.	34.283	-89.544	9/24/1993	Johnston & Haag 1996
Y.R.	Pumpkin Cr.	34.327	-89.397	5/24/1952	Suttkus et al., 1994
Y.R.	Pumpkin Cr.	34.339	-89.384	6/11/1999	Sterling et al., 2013
Y.R.	Pumpkin Cr.	34.286	-89.445	5/6/1974	Suttkus et al., 1994
Y.R.	U.T. Pumpkin Cr.	34.291	-89.44	6/30/2009	Sterling et al., 2013
Y.R.	Splinter Cr.	34.235	-89.635	10/10/2017	S. Adams, 2017, person. comm.
Y.R.	Splinter Cr.	34.25	-89.642	10/10/2017	S. Adams, 2017, person. comm.
Y.R.	Tarvers Cr.	34.251	-89.489	5/10/2018	A. Carson, 2018, person. comm.
Y.R.	Taylor Cr.	34.271	-89.58	8/20/1991	Ross et al., 2001
Y.R.	Taylor Cr.	34.296	-89.588	3/23/2018	K. Sterling, 2018, person. observ.
Y.R.	U.T. Taylor Cr.	34.123	-89.641	5/11/1988	Suttkus et al., 1994
Y.R.	U.T. Taylor Cr.	34.293	-89.588	3/23/2018	K. Sterling, 2018, person. observ.
Y.R.	Yellow Leaf Cr.	34.375	-89.421	6/9/1999	Sterling et al., 2013
Y.R.	Yellow Leaf Cr.	34.379	-89.413	6/9/1999	Sterling et al., 2013
Y.R.	Yellow Leaf Cr.	34.396	-89.404	4/12/1988	Ross et al., 2001

Y.R.	Yellow Leaf Cr.	34.368	-89.429	summer 2012	M. Bland, 2019, person. communication
Y.R.	U.T. Yocona River	34.273	-89.412	summer 2012	M. Bland, 2019, person. communication

Male nuptial color photographs*:* All fishes were photographed immediately after capture and then released (except for six type specimen vouchers). The photographs are presented in the same order with the same labels as were shown to colleagues for color scoring (see Methods section). A key (after the photographs) indicates which drainage individuals were sampled from.

Male nuptial color photographs; credit for all photographs: Ken Sterling

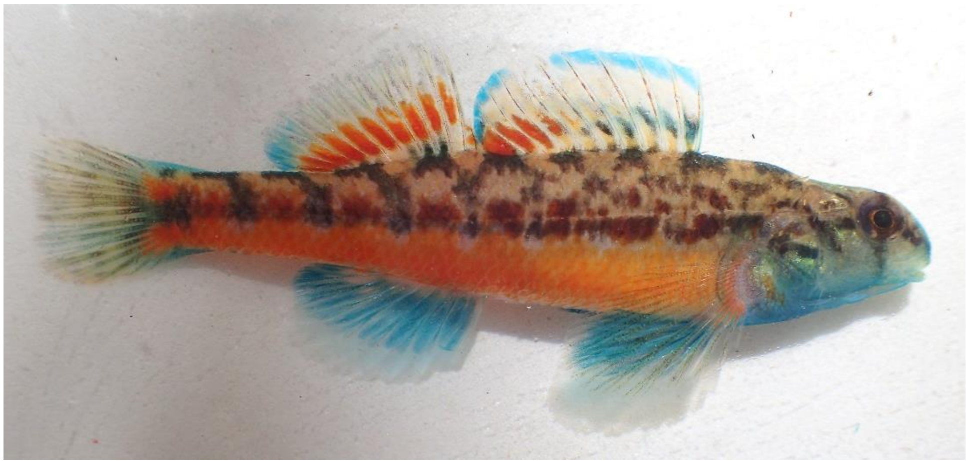

YD_1

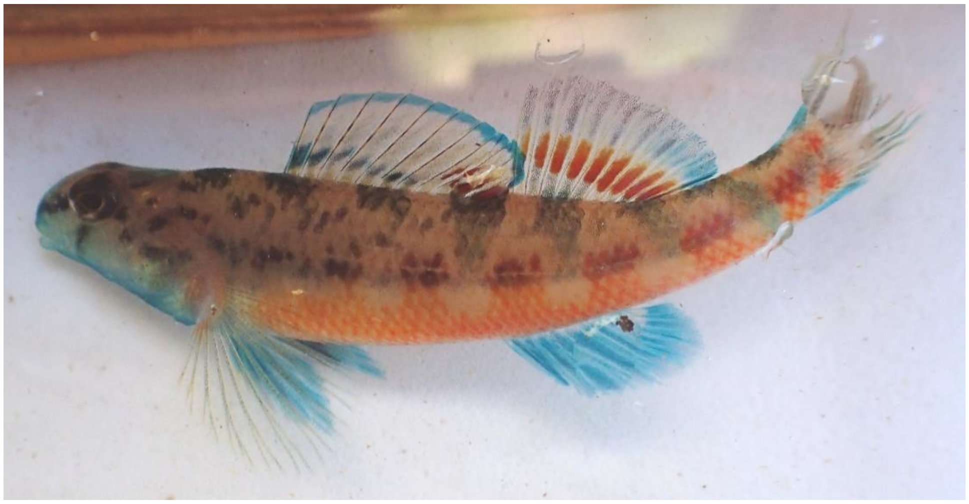

YD_2

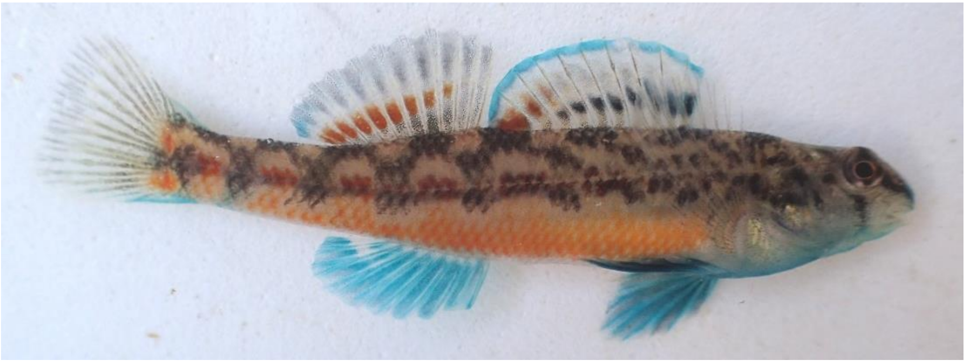

YD_3

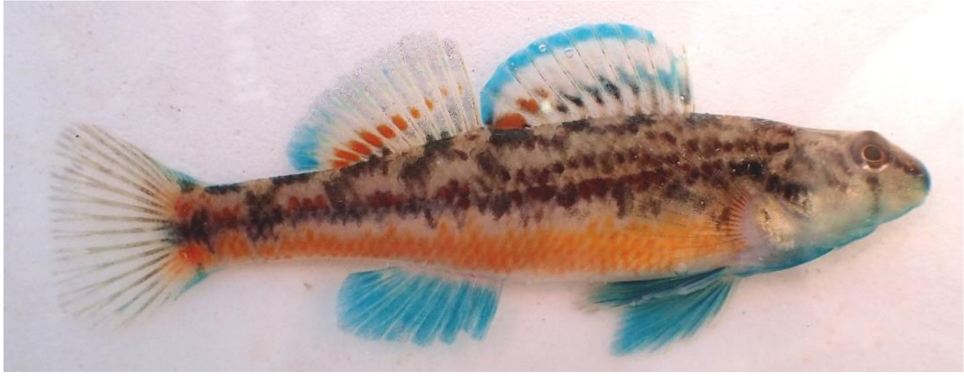

YD_4

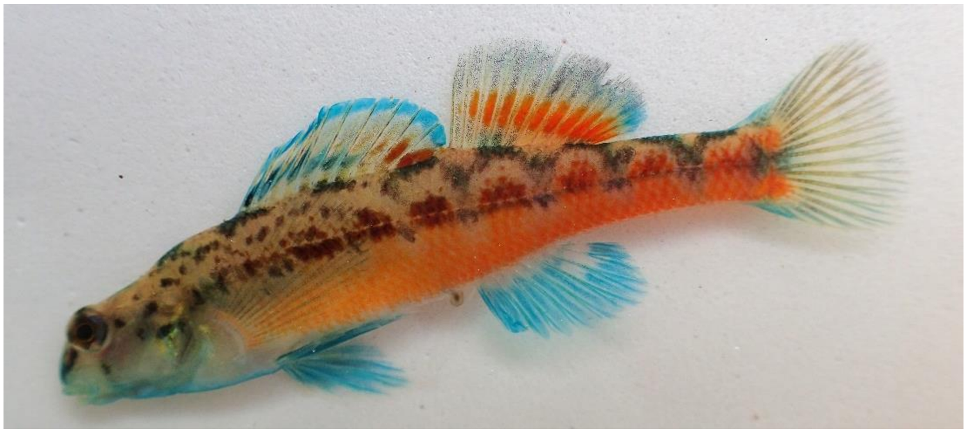

YD_5

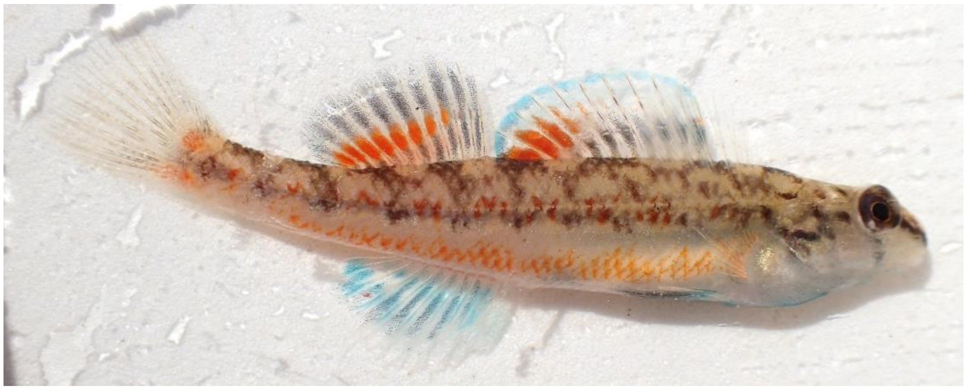

YD_6

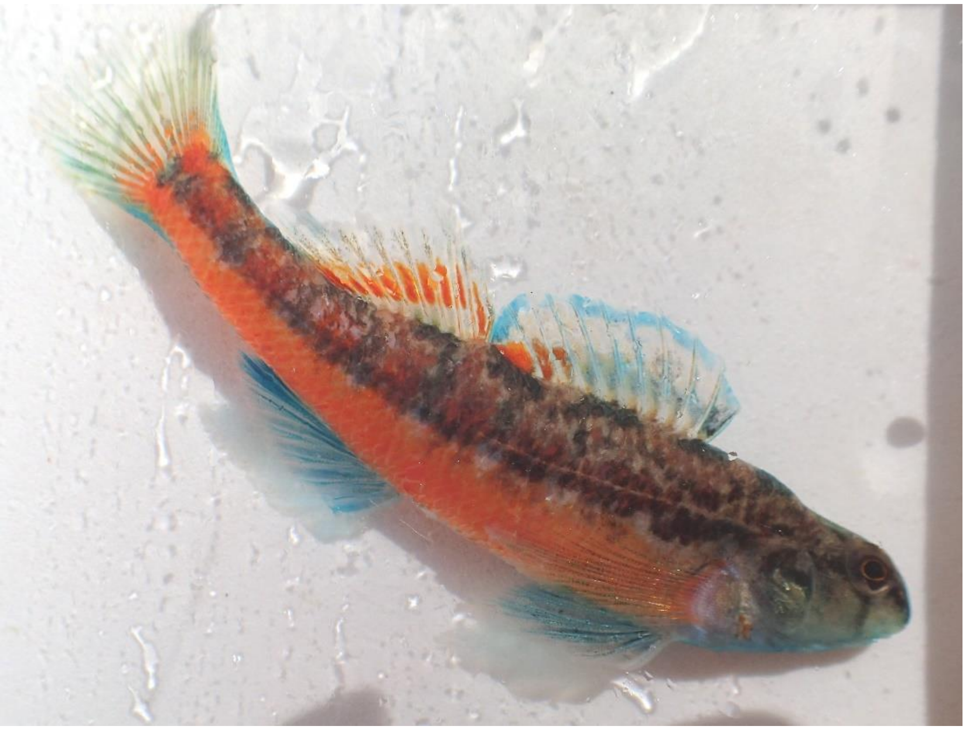

YD_7

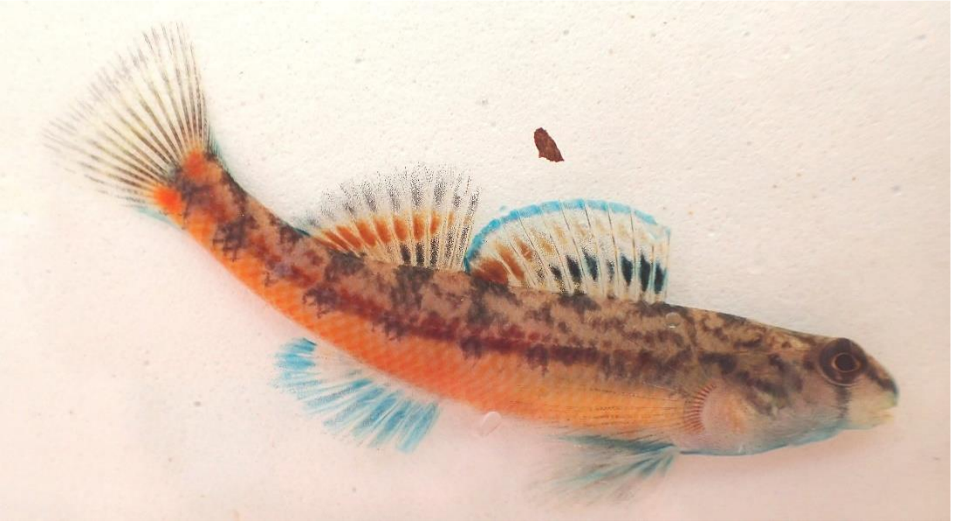

YD_8

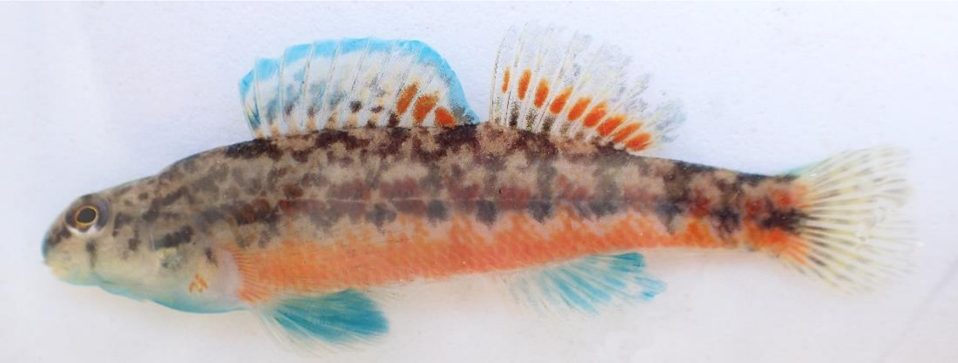

YD_9

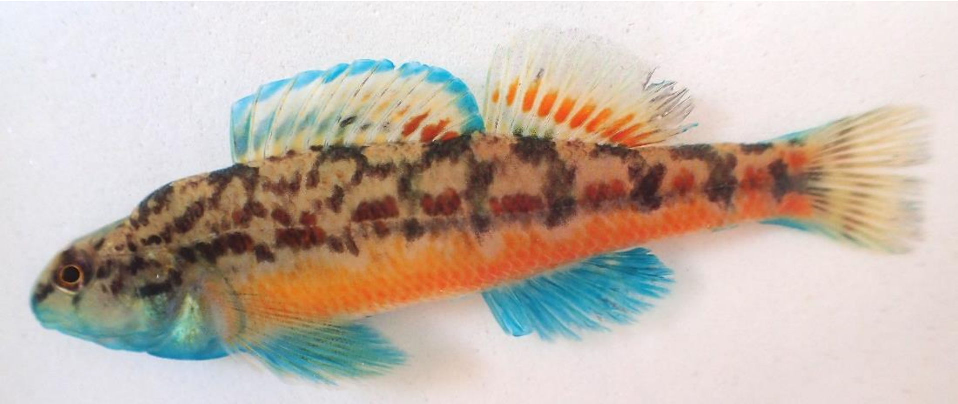

YD_10

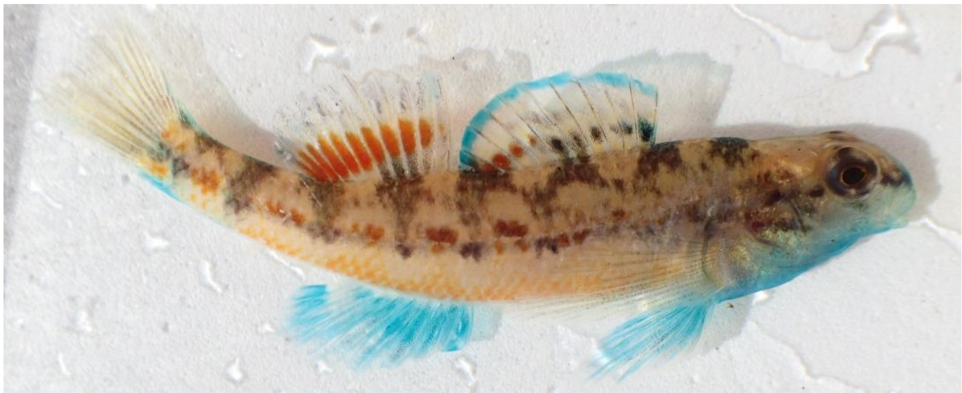

YD_11

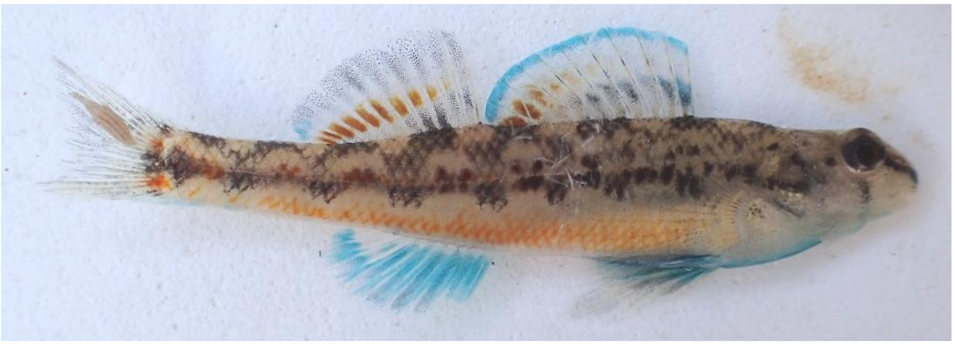

YD_12

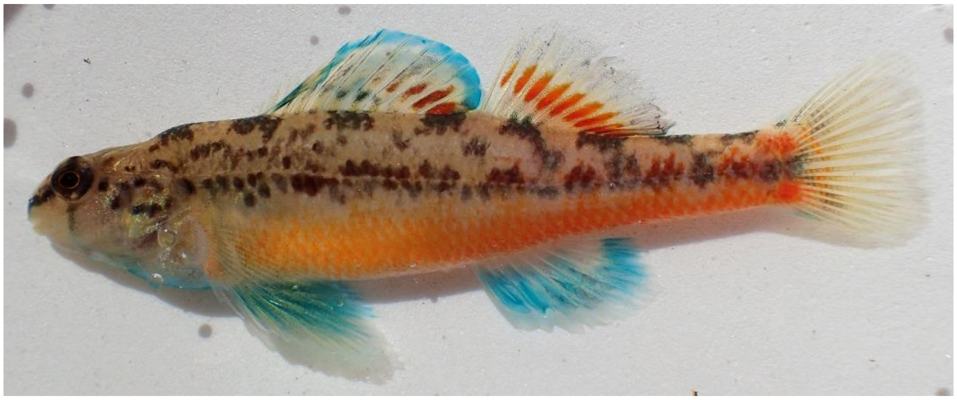

YD_13

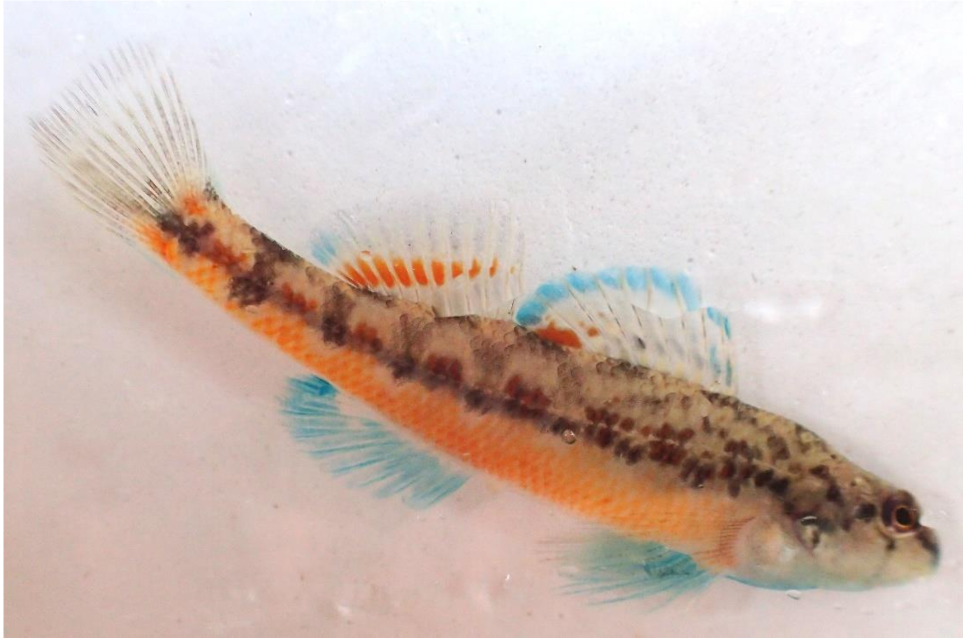

YD_14

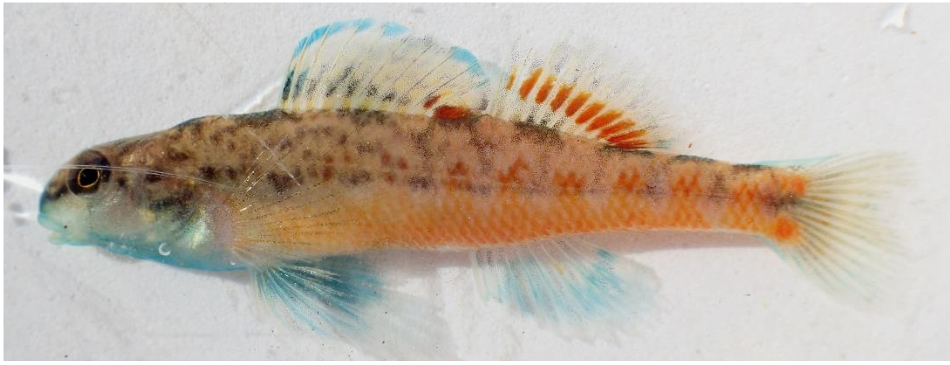

YD_15

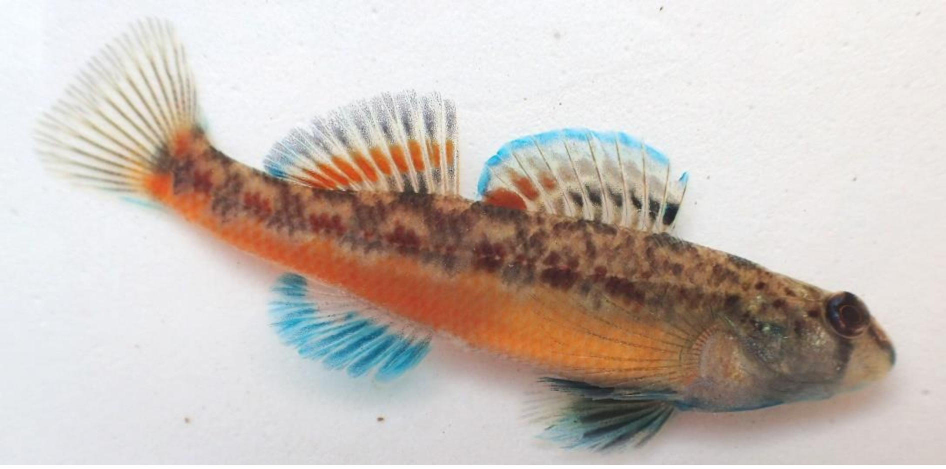

YD_16

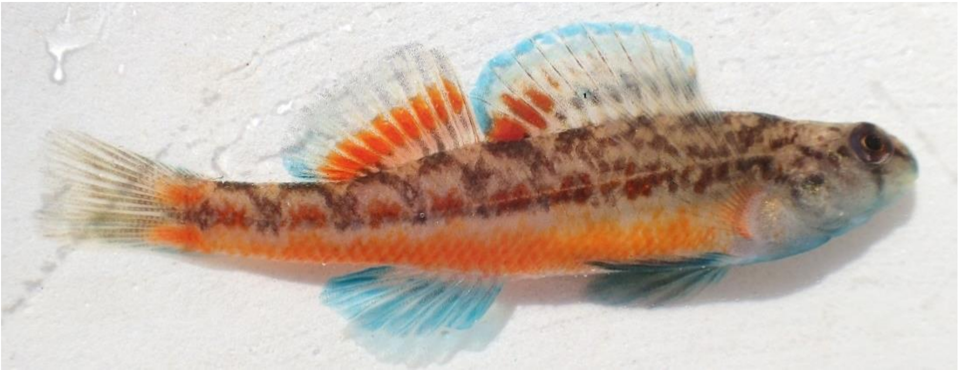

YD_17

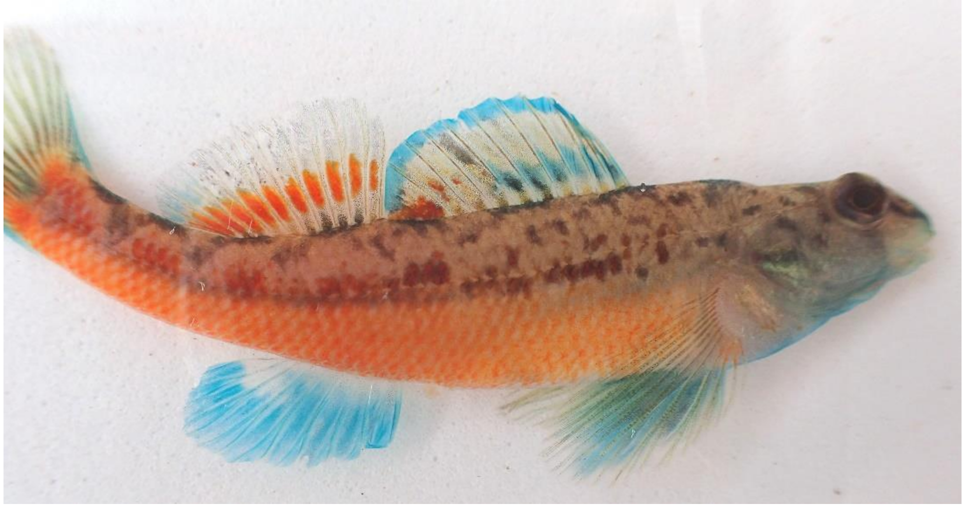

YD_18

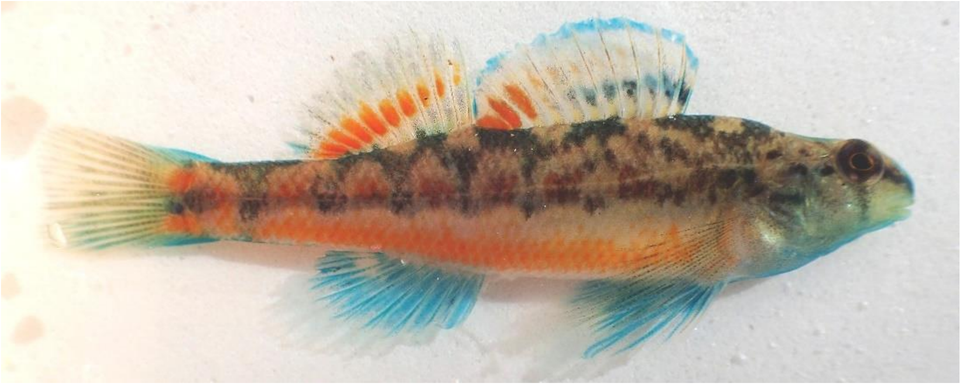

YD_19

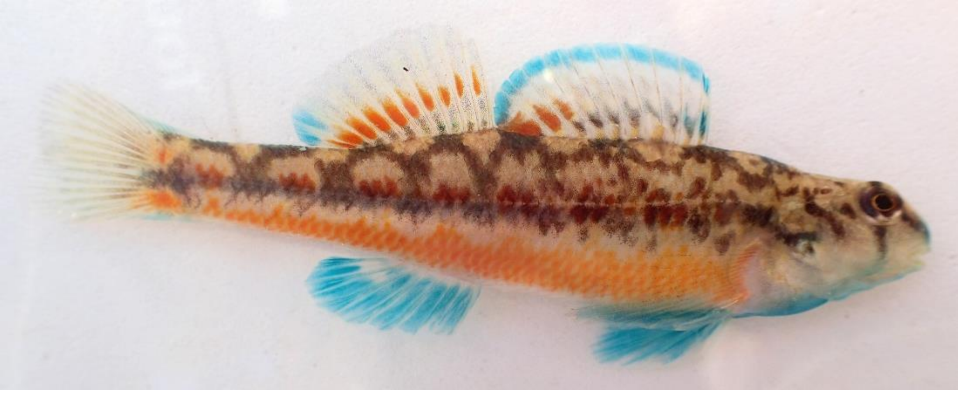

YD_20

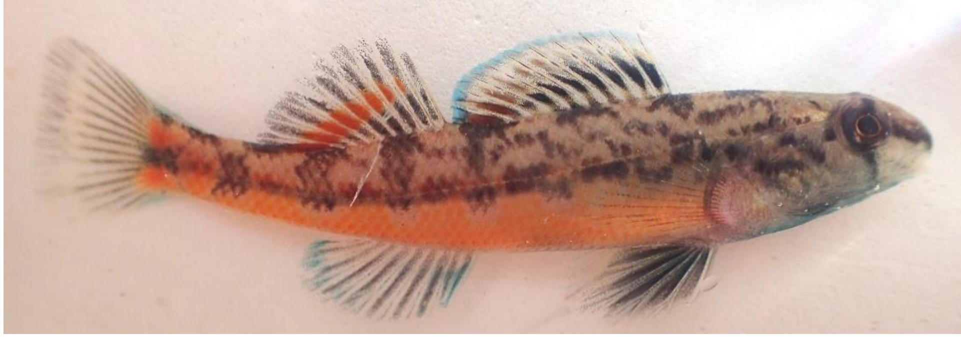

YD_21

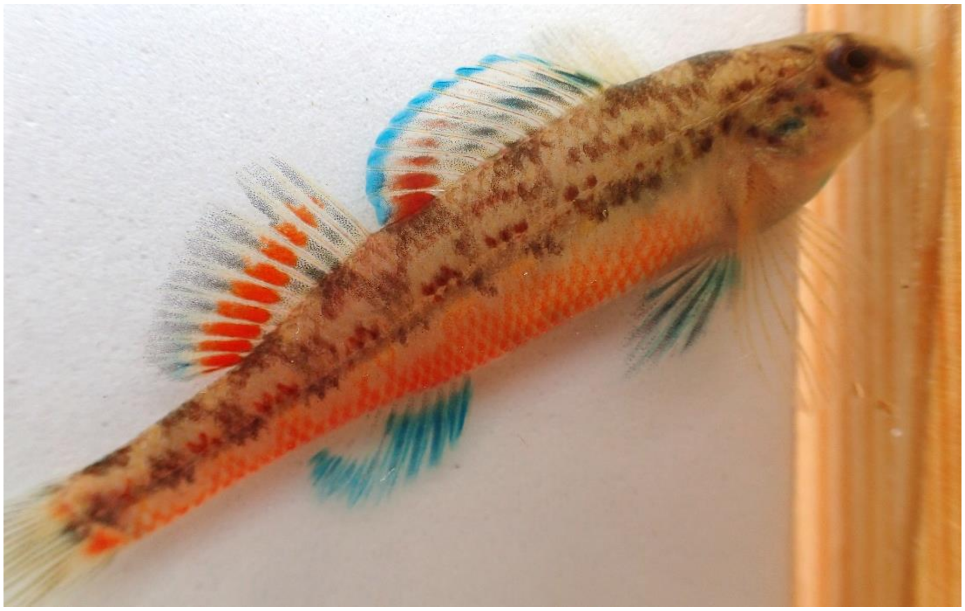

YD_22

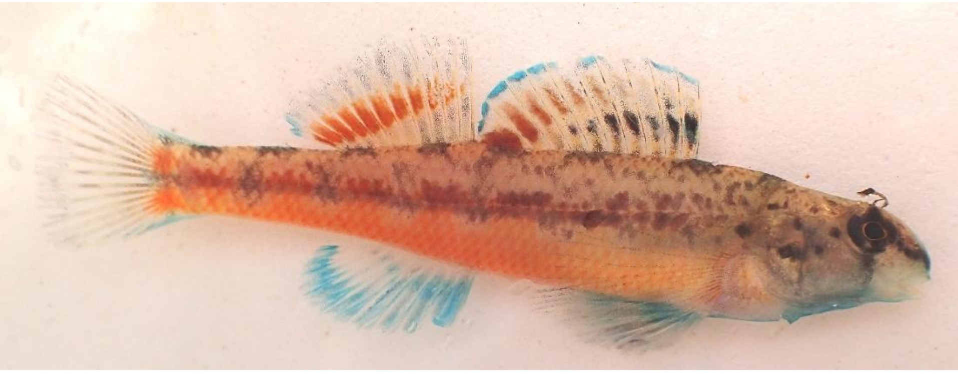

YD_23

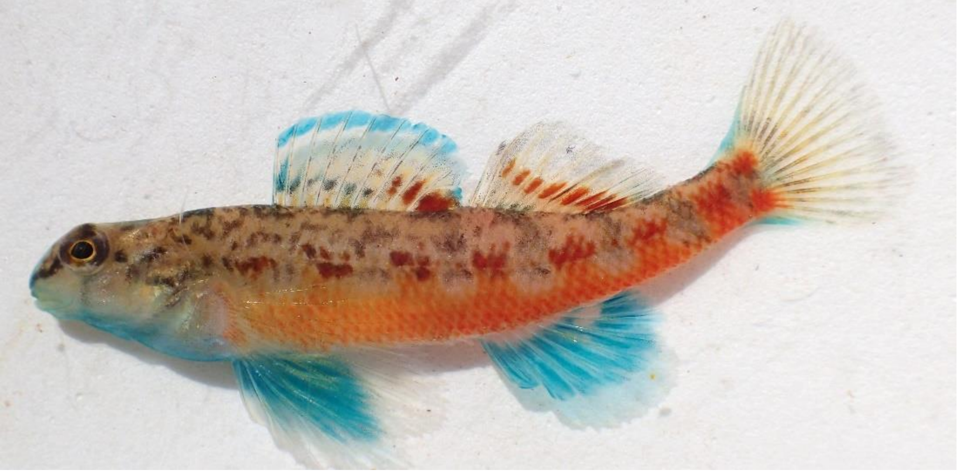

YD_24

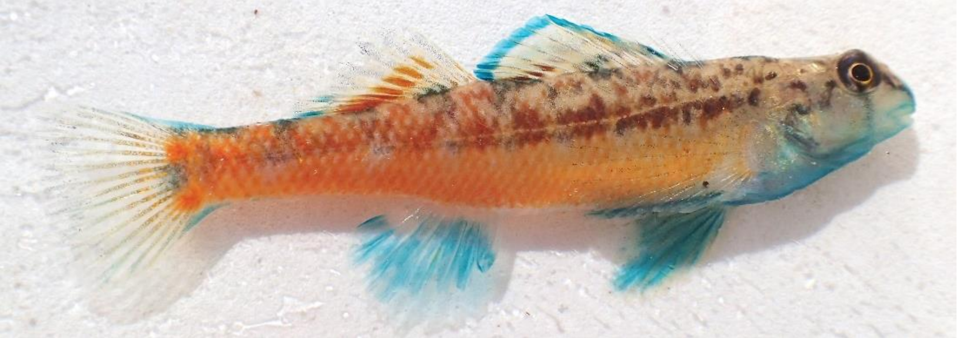

YD_25

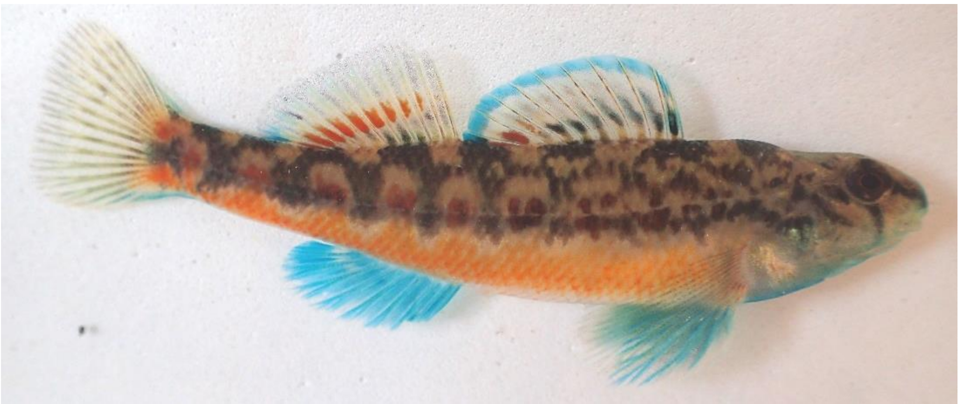

YD_26

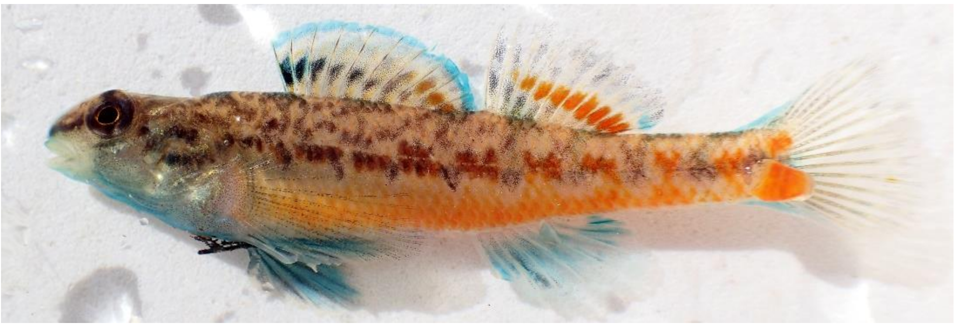

YD_27

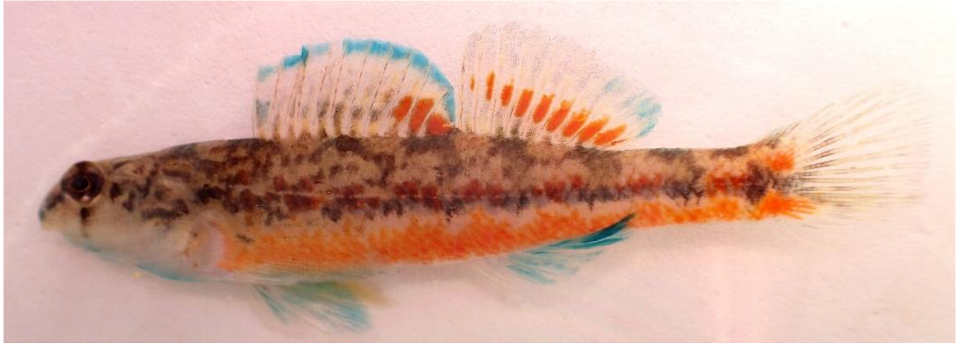

YD_28

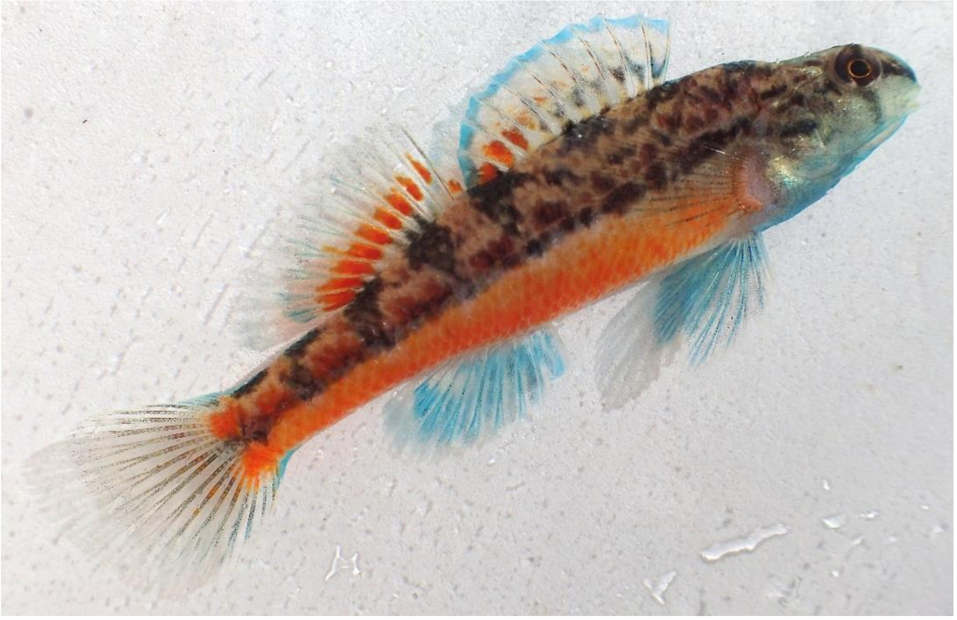

YD_29

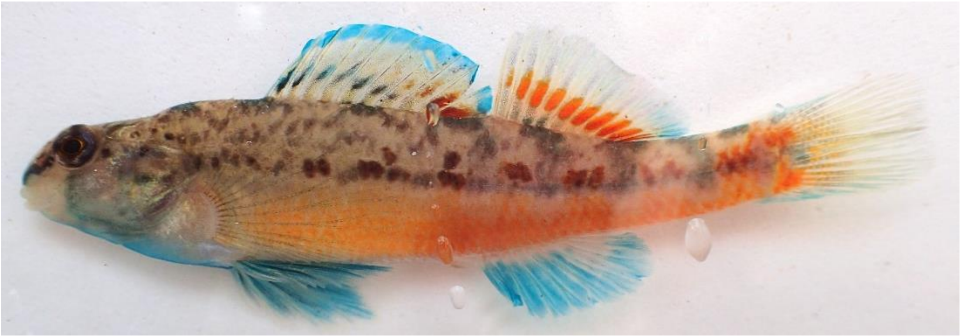

YD_30

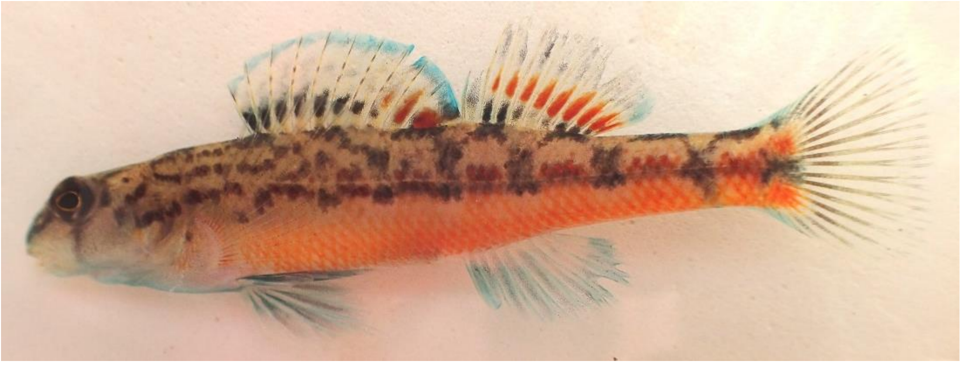

YD_31

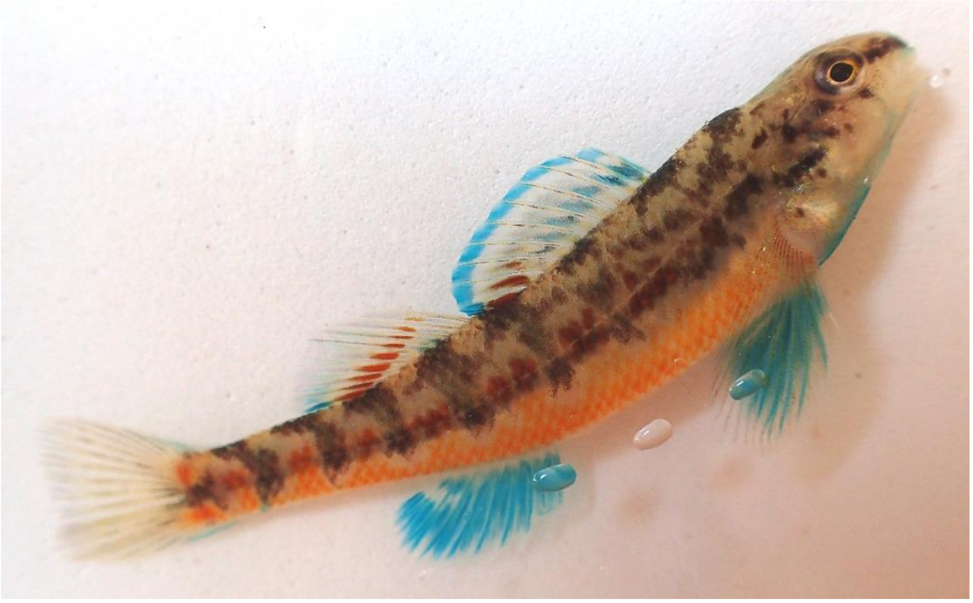

YD_32

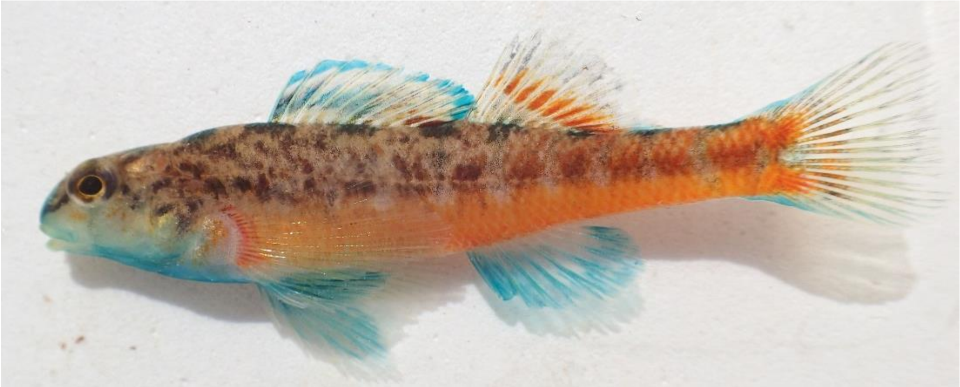

YD_33

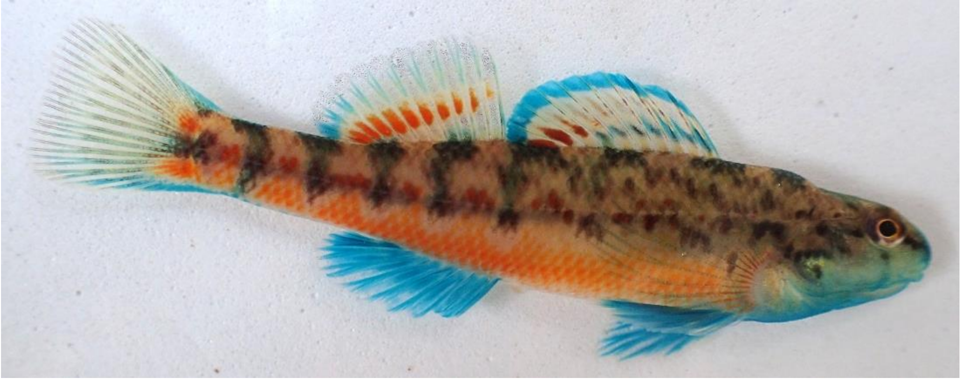

YD_34

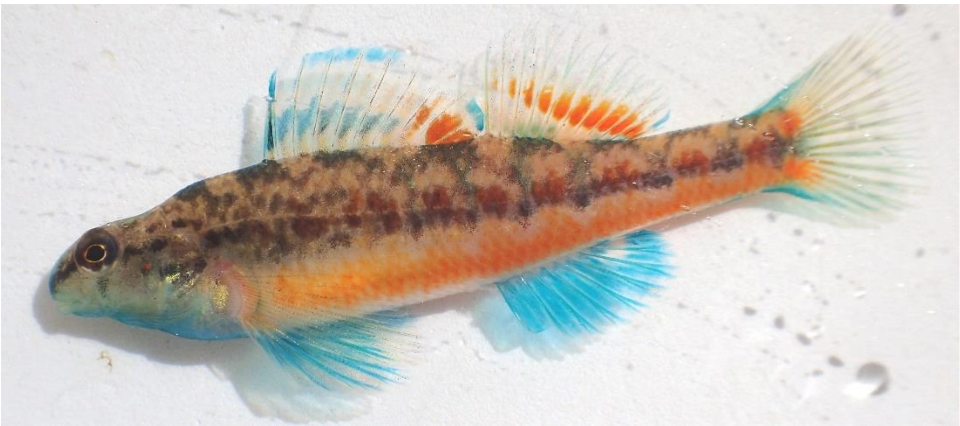

YD_35

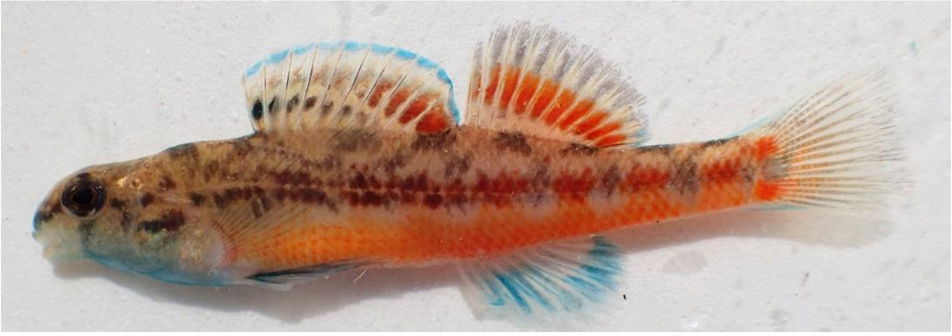

YD_36

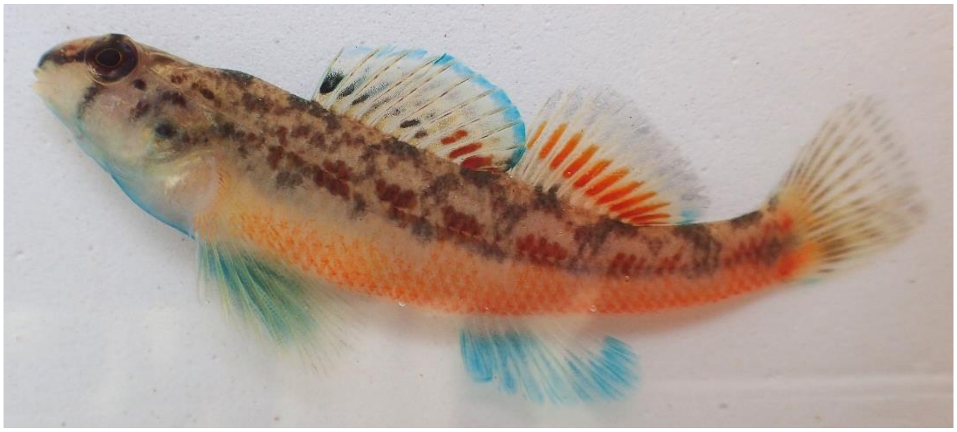

YD_37

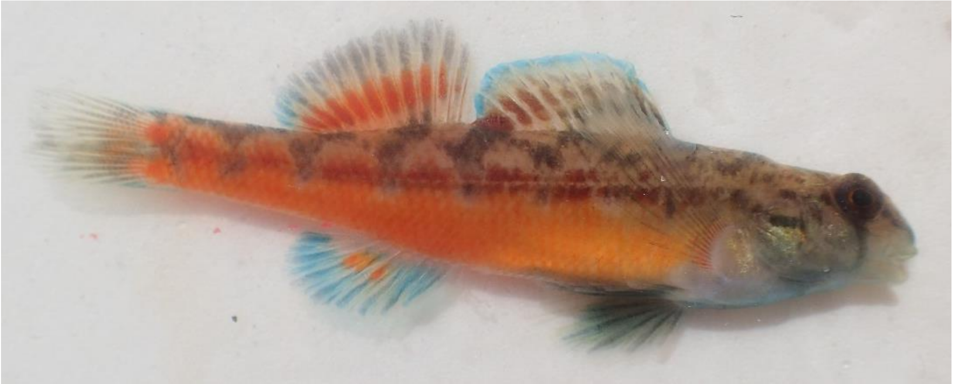

YD_38

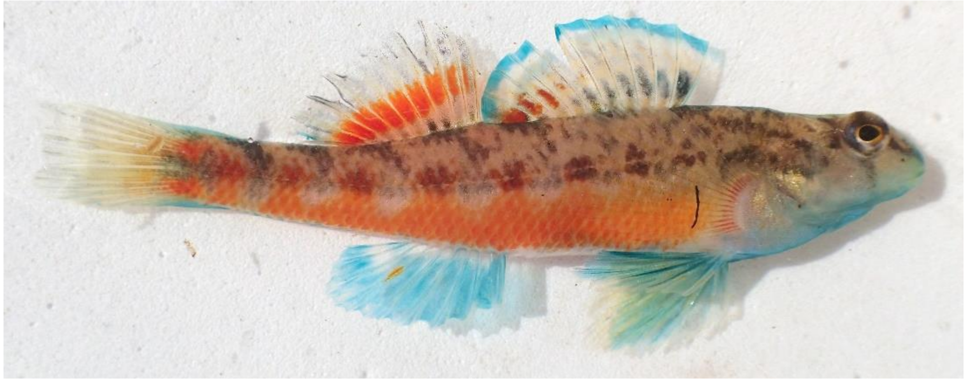

YD_39

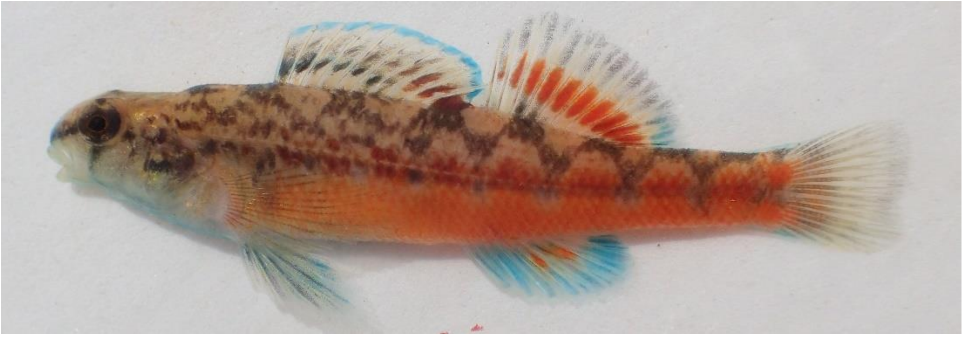

YD_40

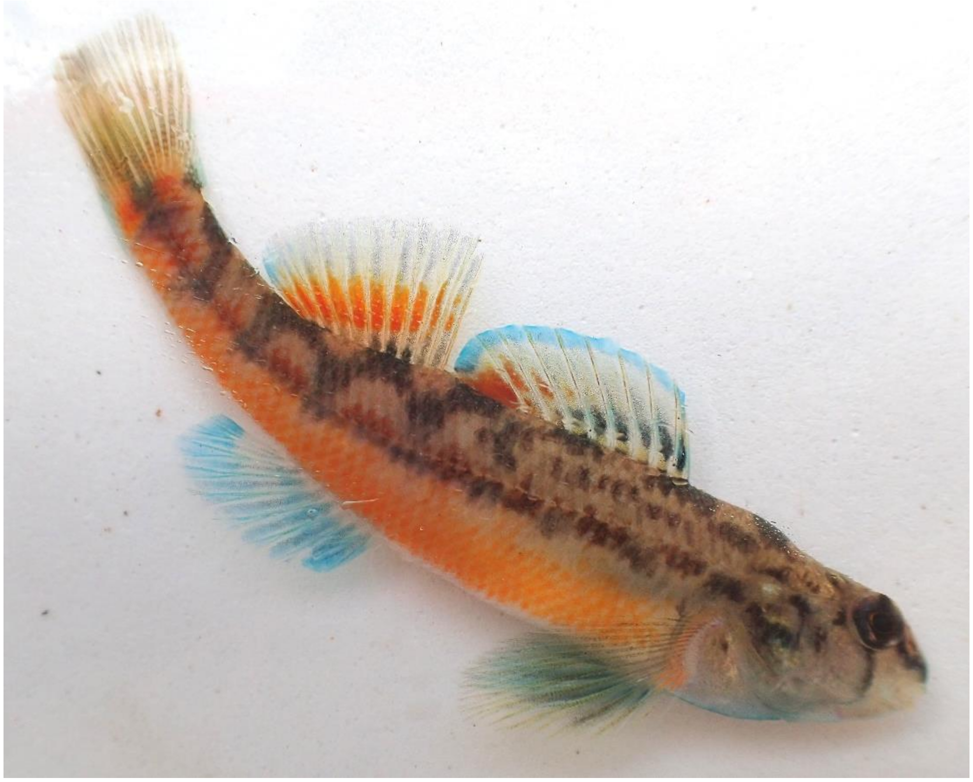

YD_41

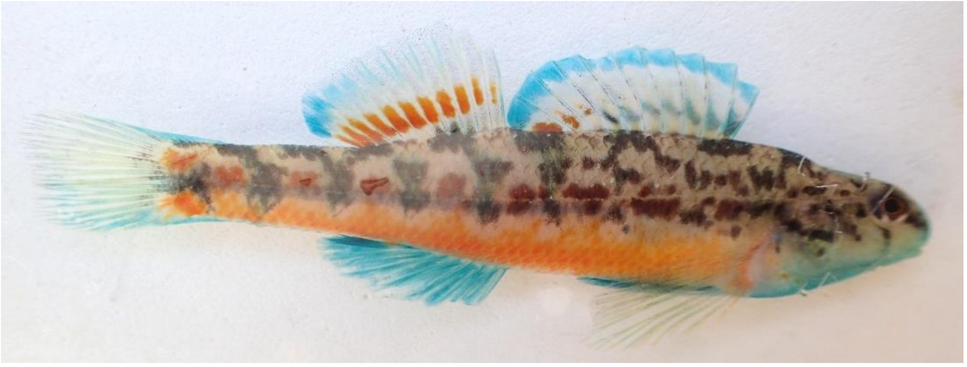

YD_42

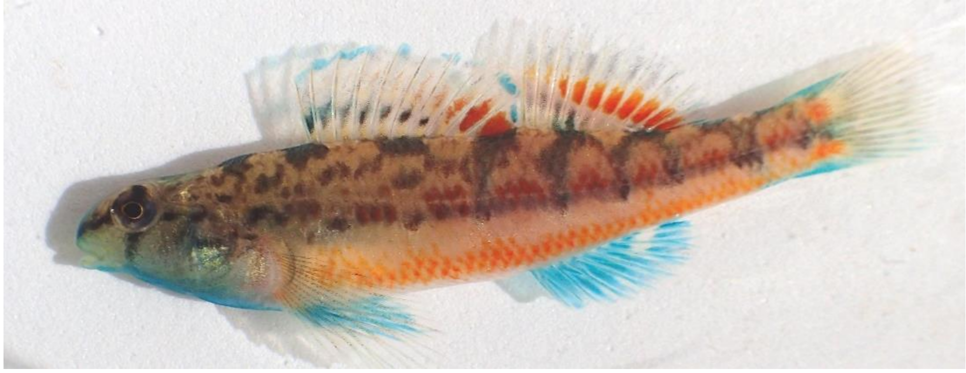

YD_43

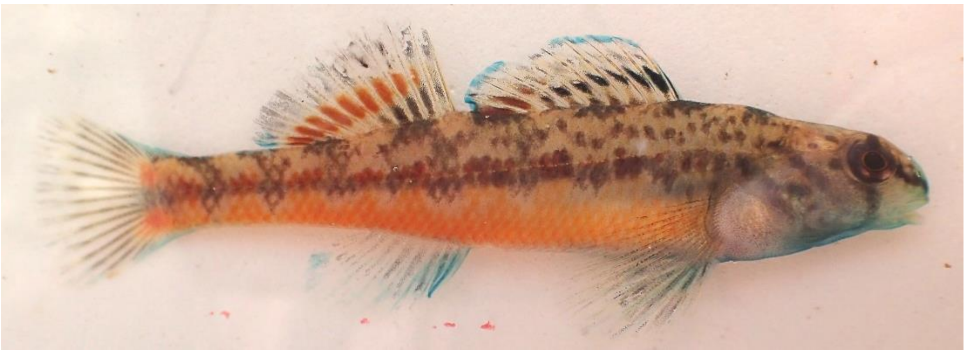

YD_44

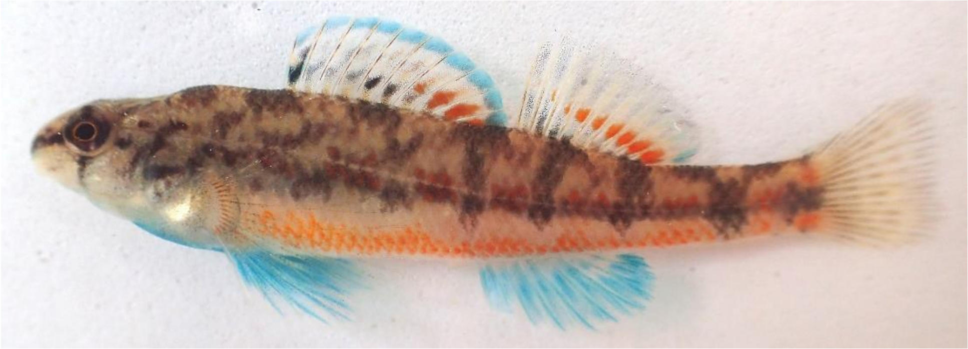

YD_45

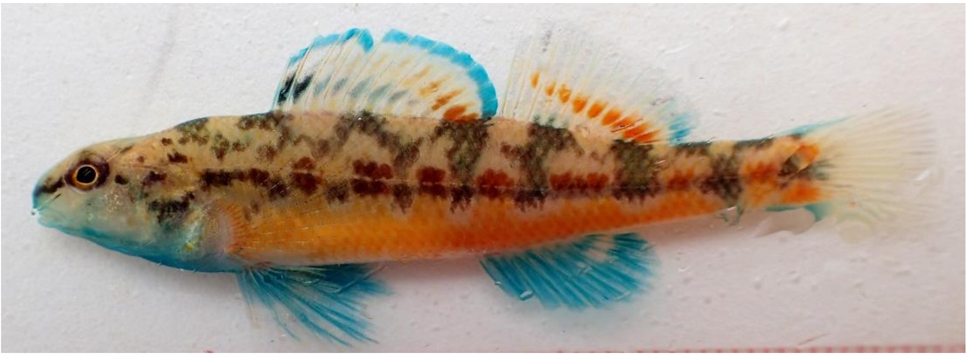

YD_46

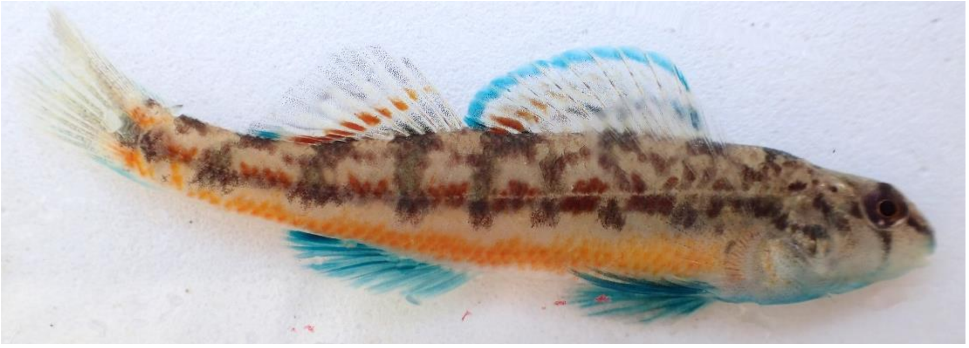

YD_47

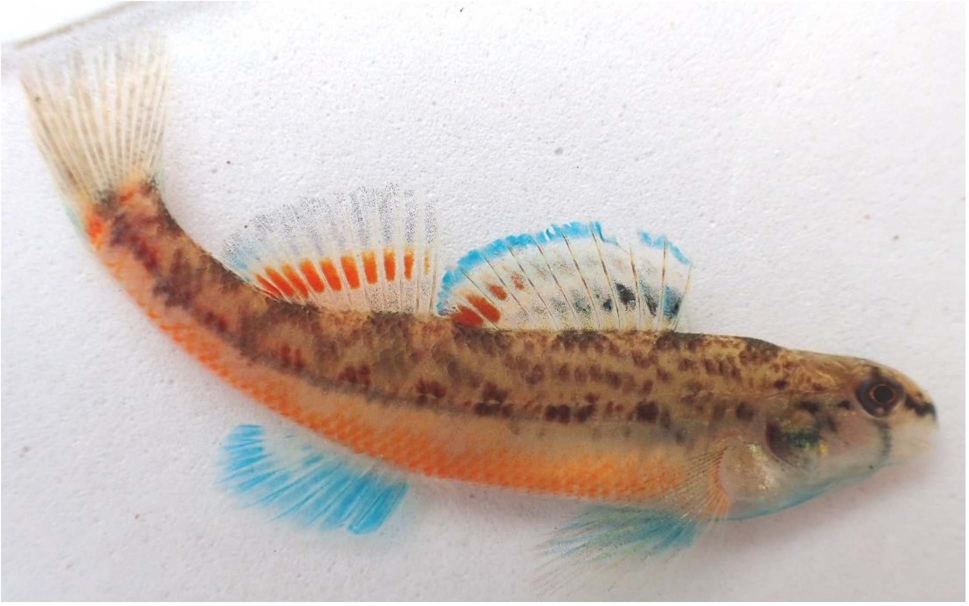

YD_48

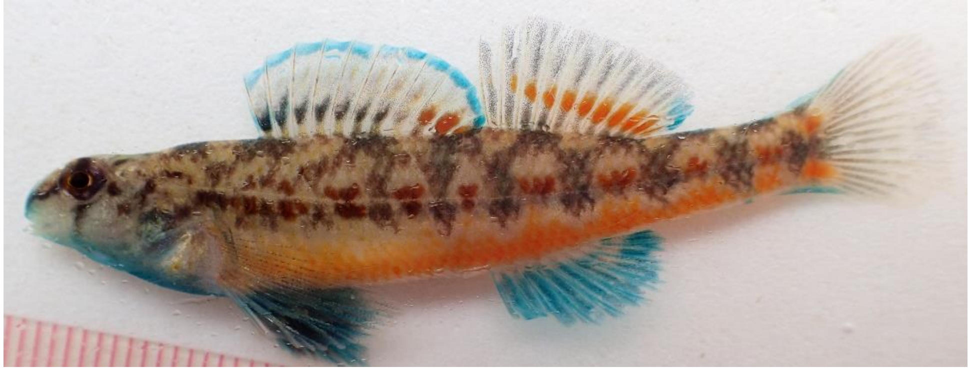

YD_49

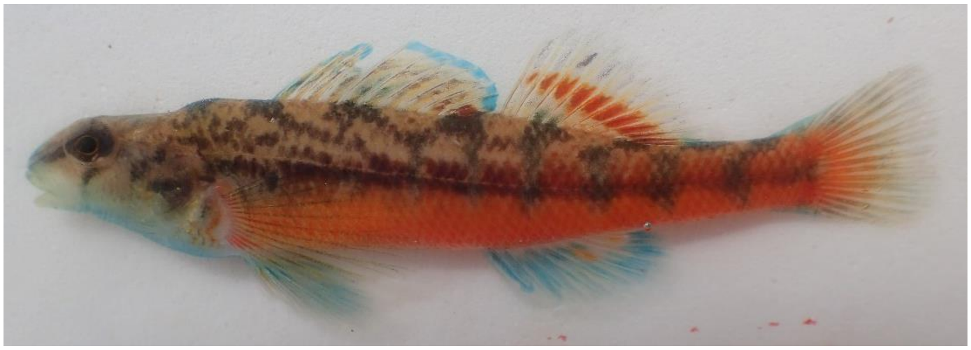

YD_50

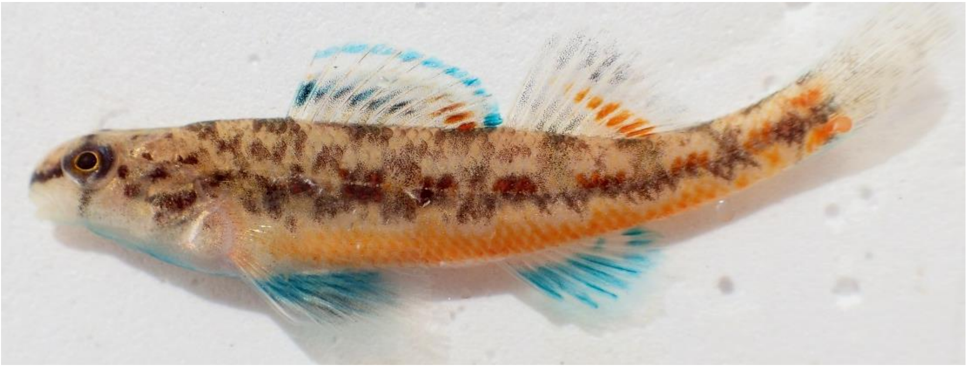

YD_51

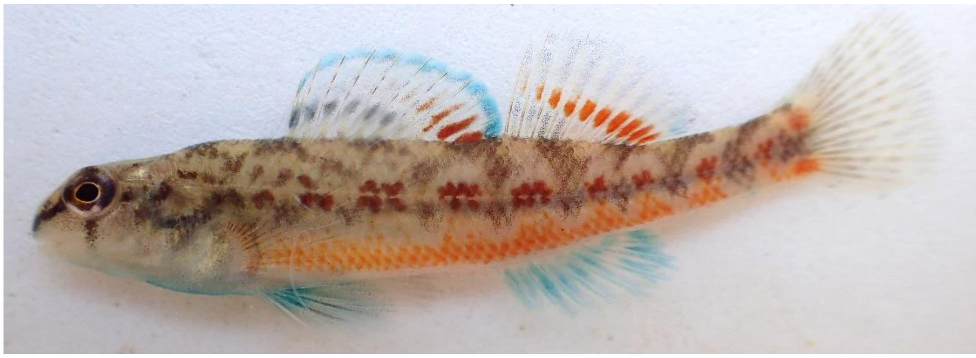

YD_52

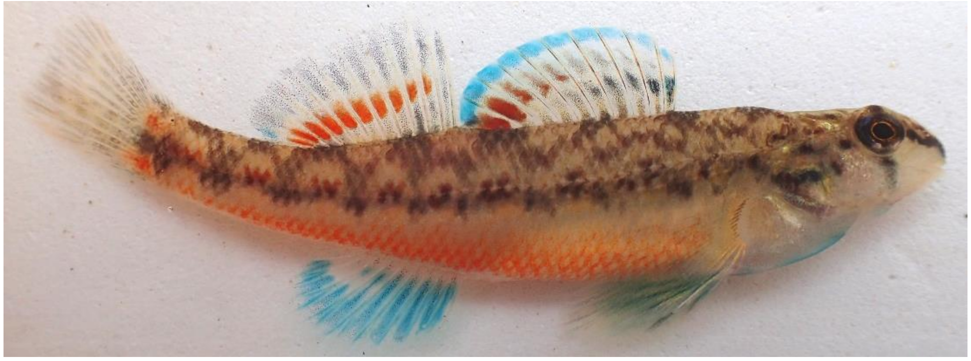

YD_53

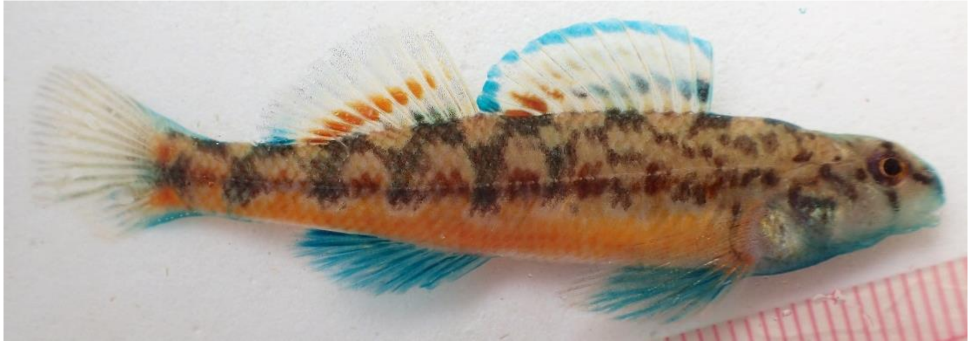

YD_54

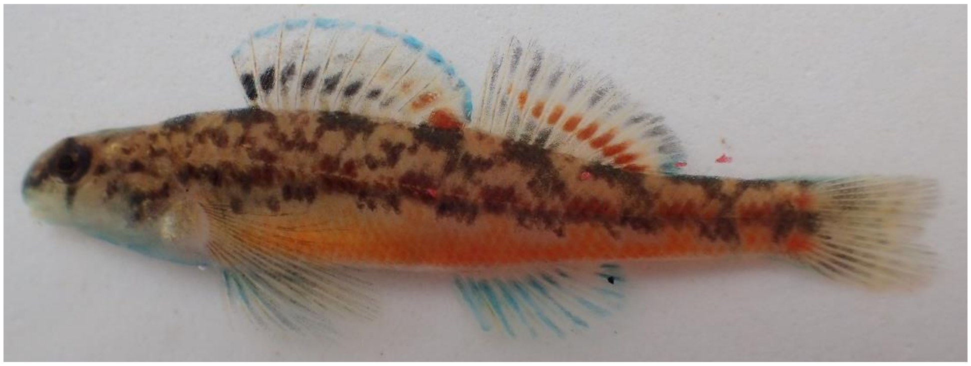

YD_55

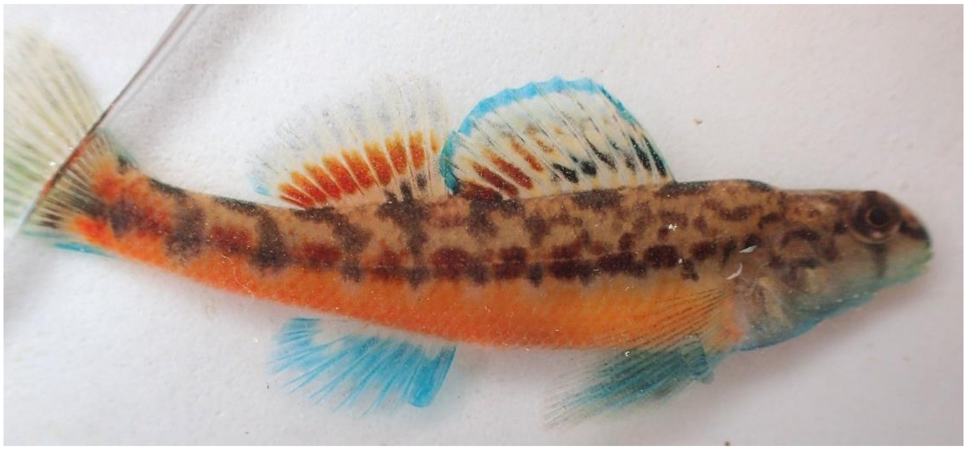

YD_56

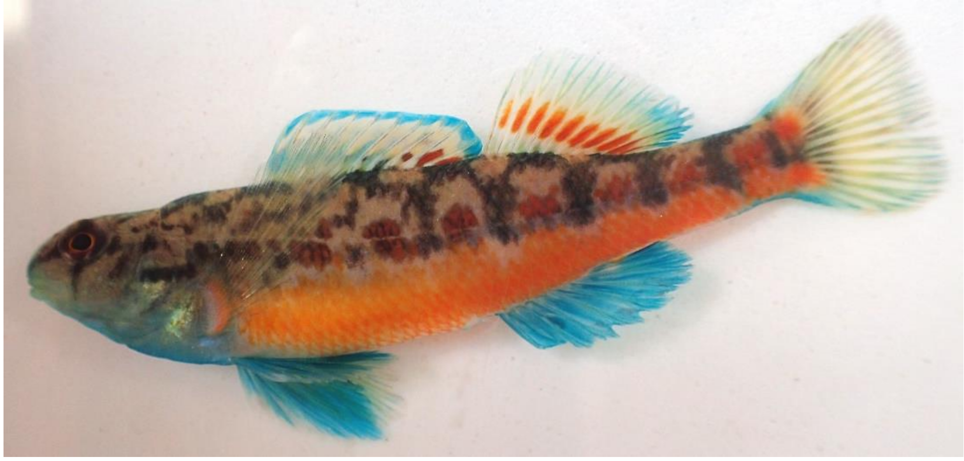

YD_57

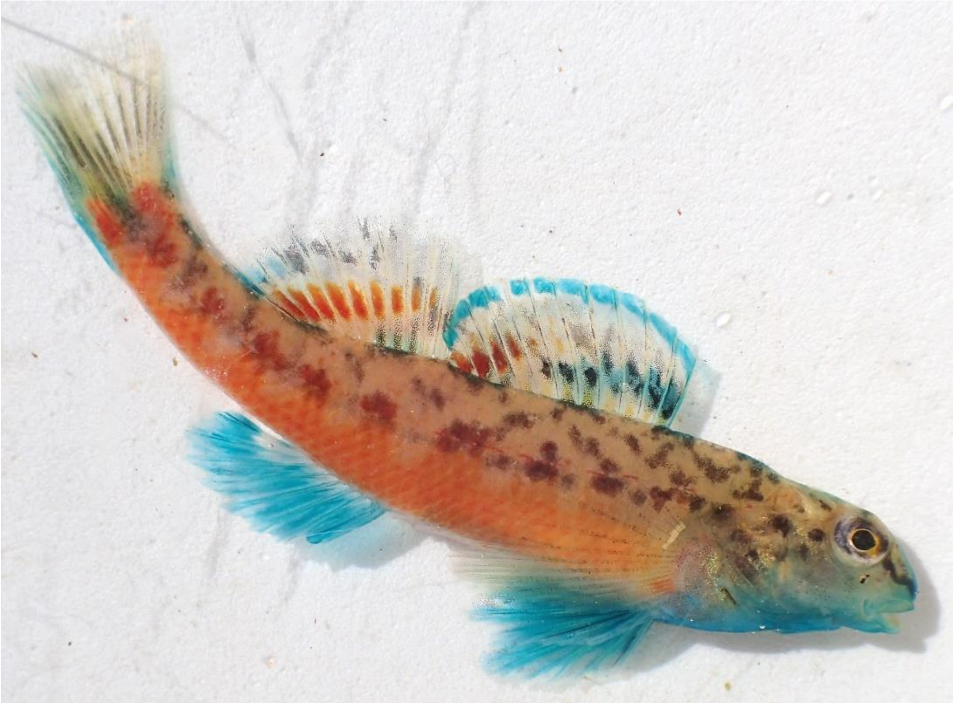

YD_58

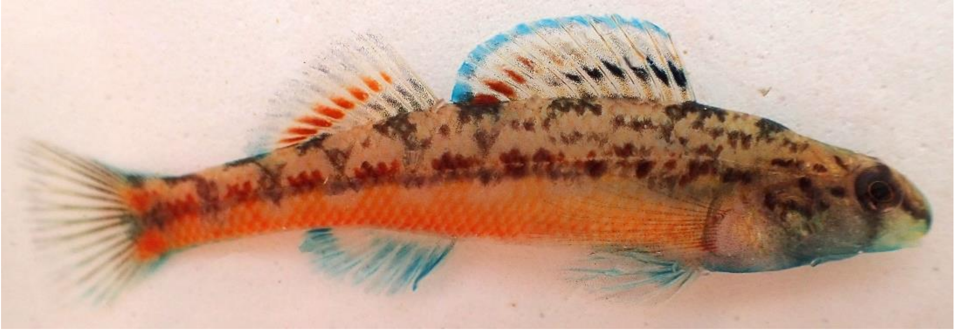

YD_59

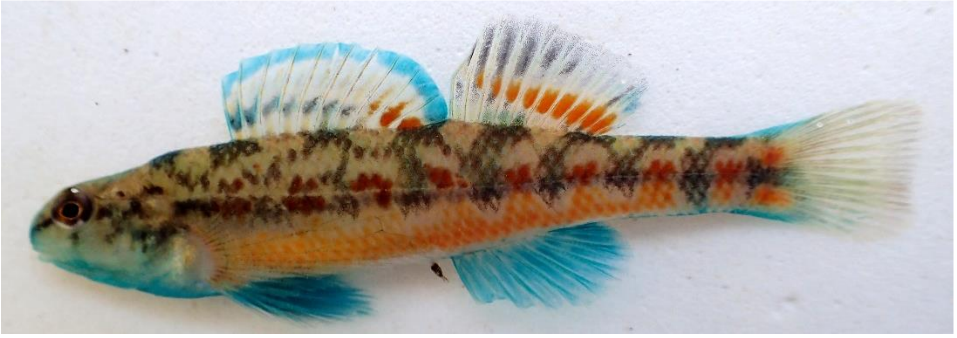

YD_60

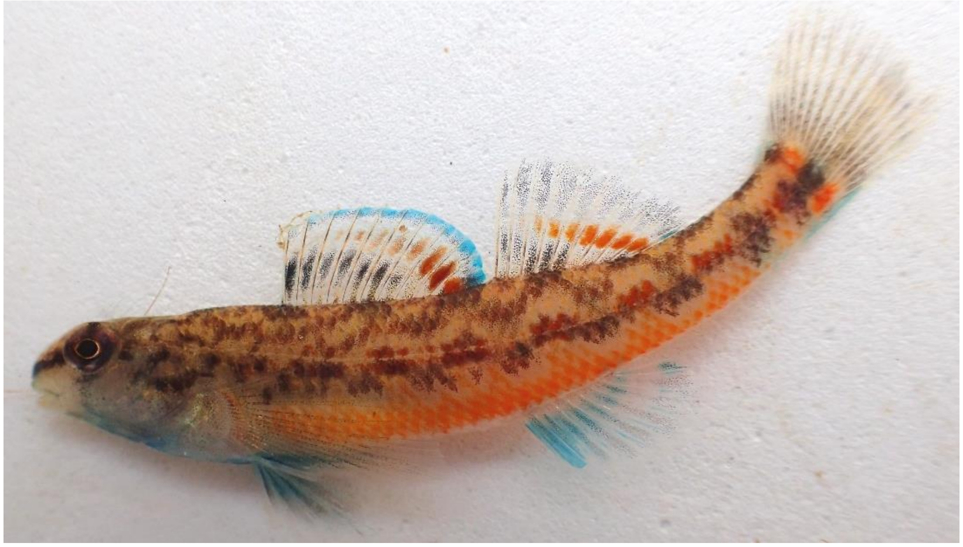

YD_61

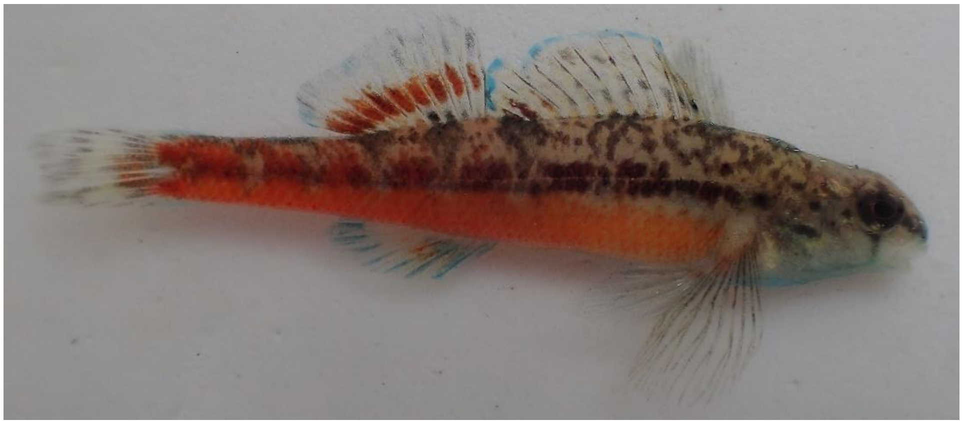

YD_62

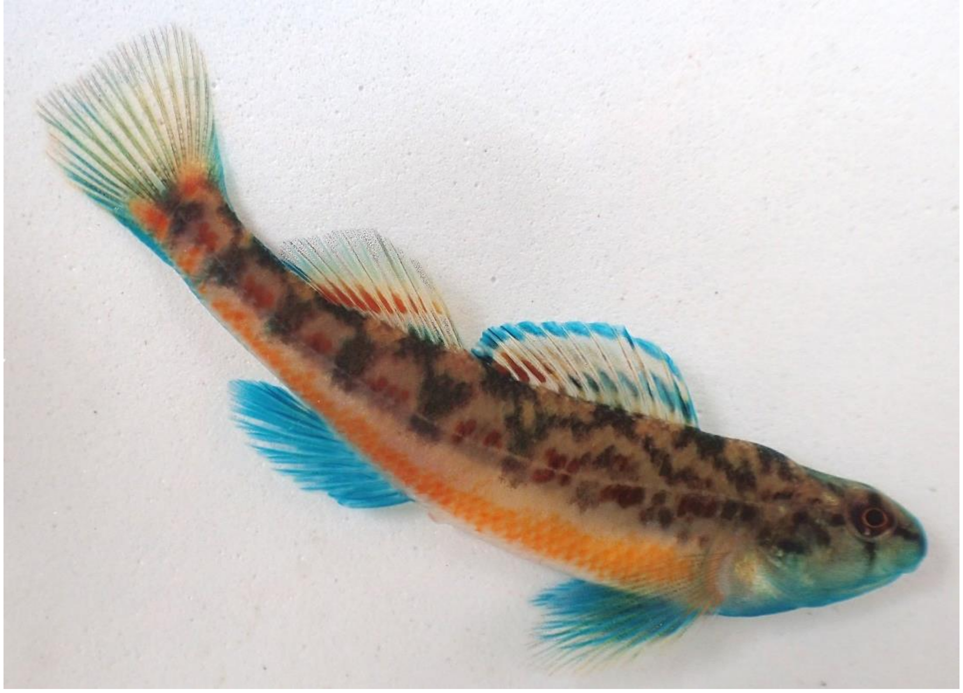

YD_63

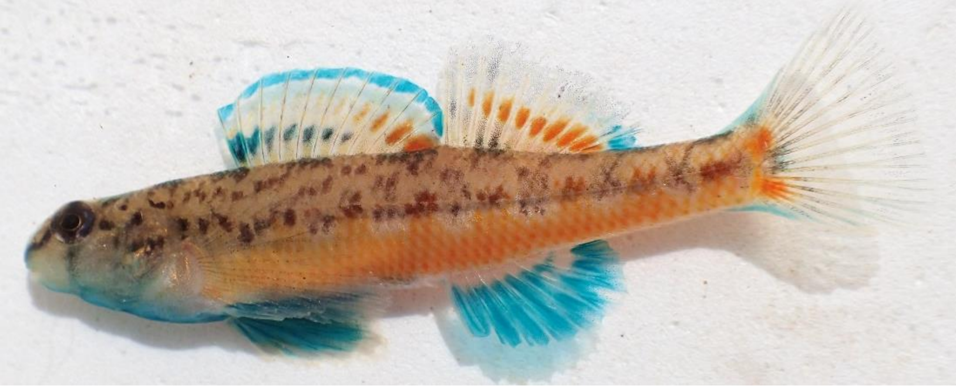

YD_64

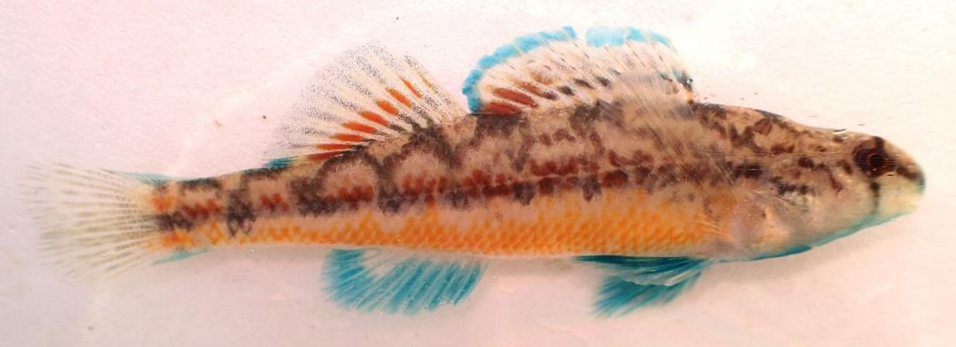

YD_65

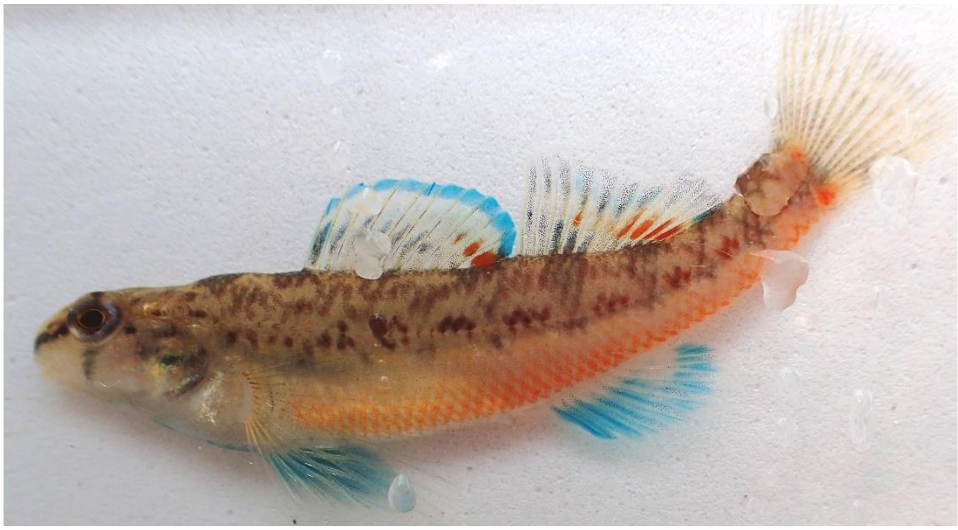

YD_66

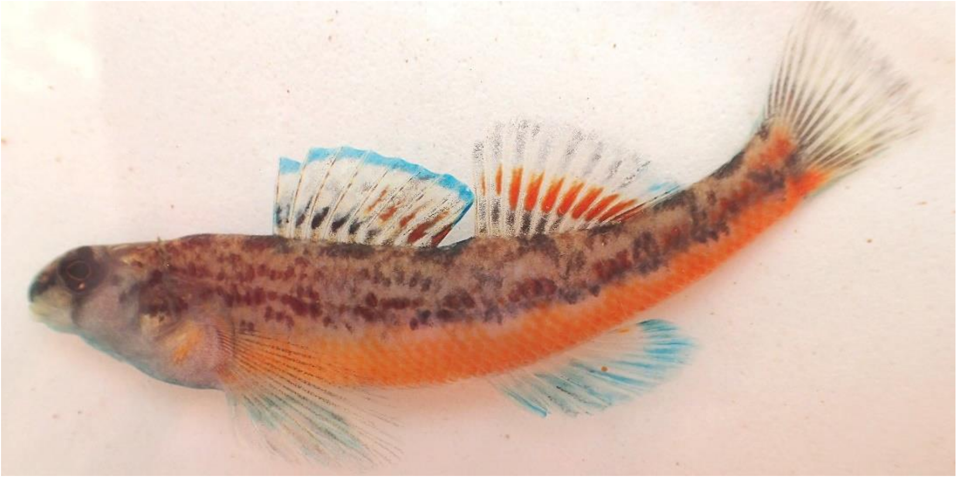

YD_67

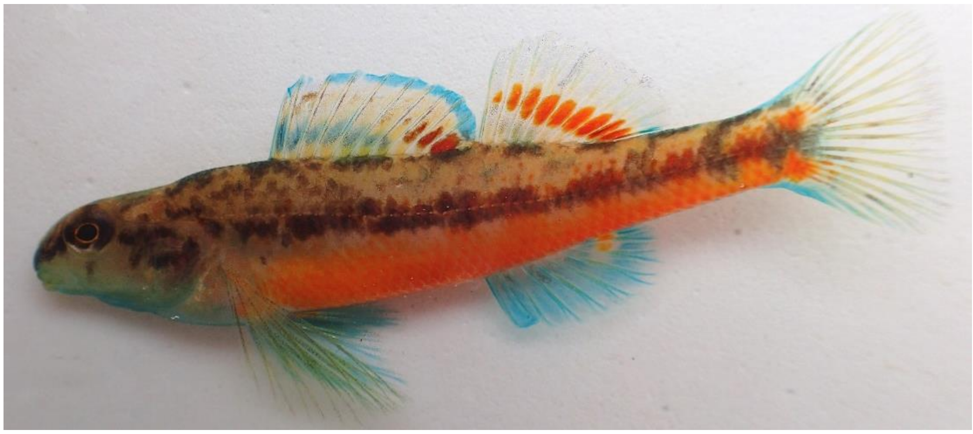

YD_68

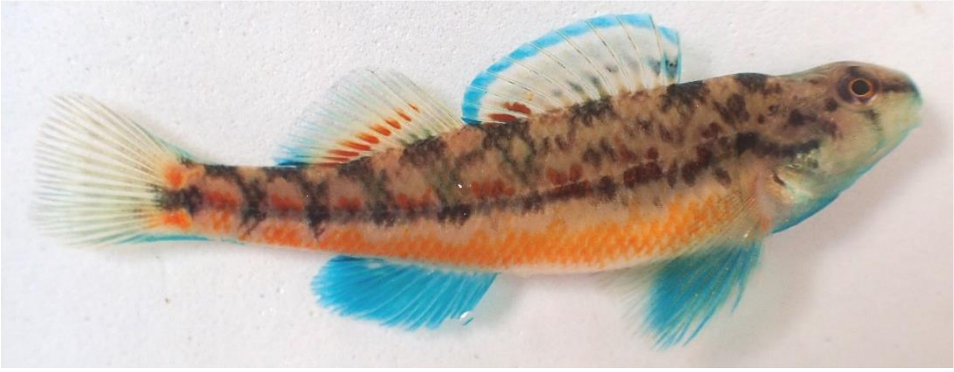

YD_69

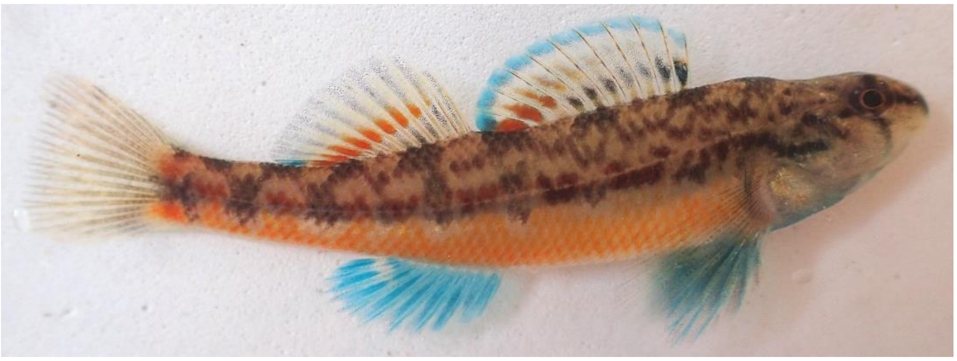

YD_70

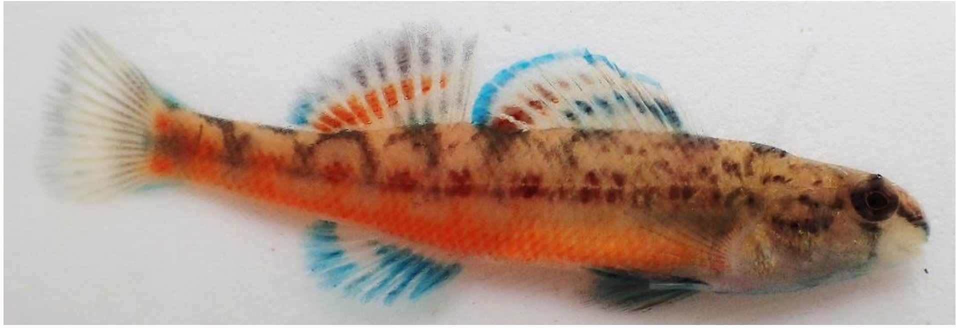

YD_71

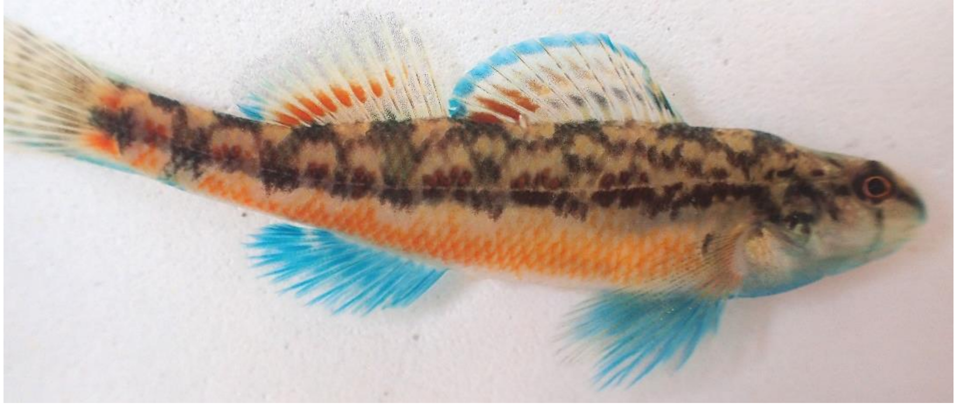

YD_72

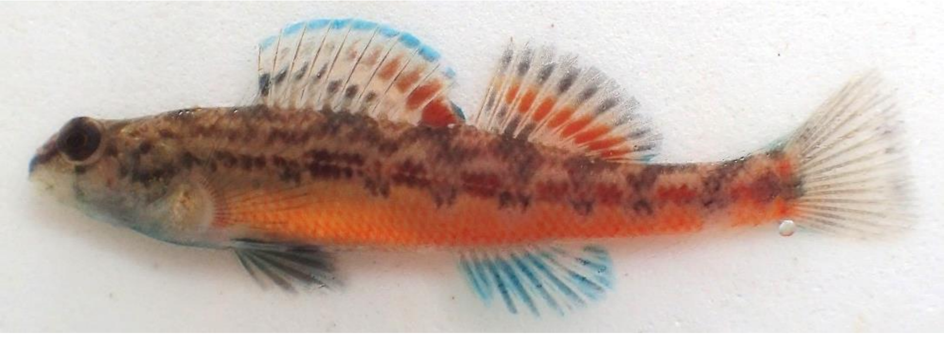

YD_73

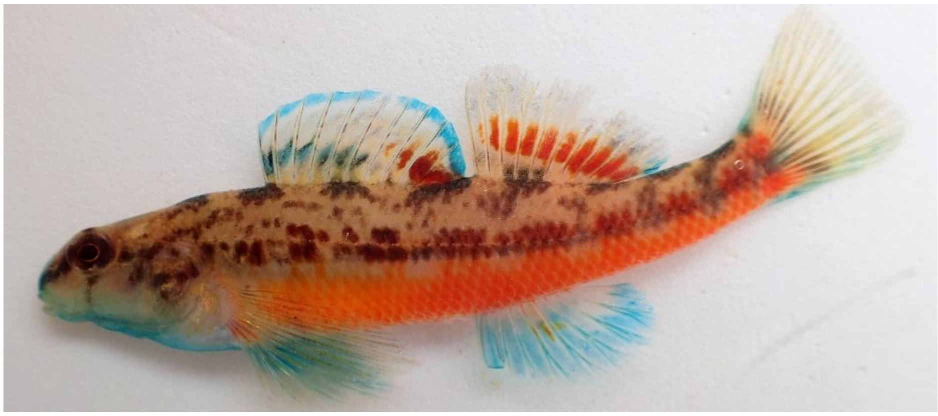

YD_74

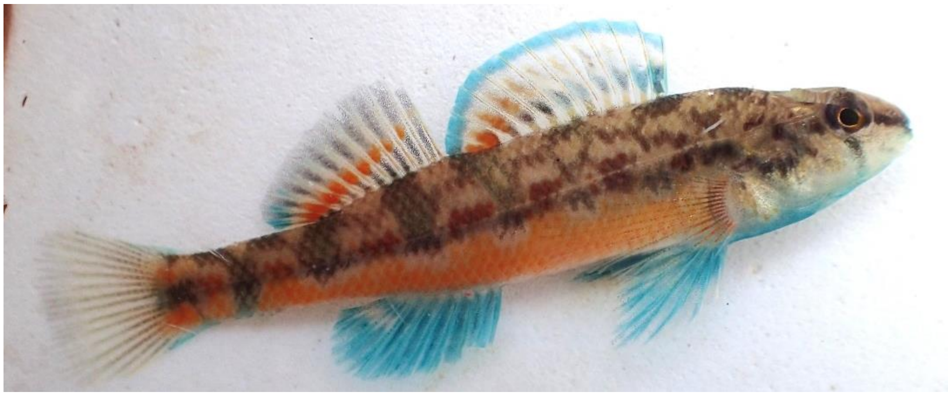

YD_75

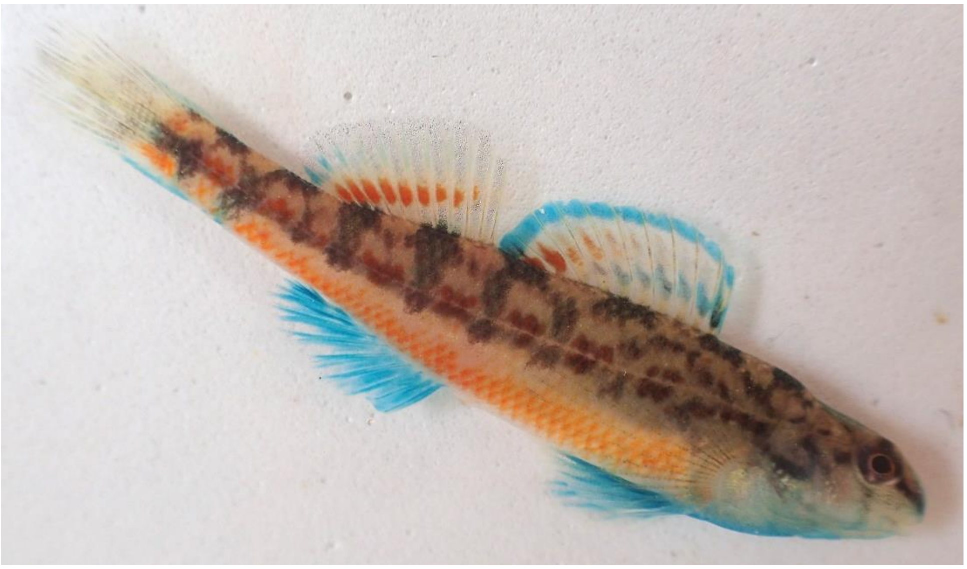

YD_76

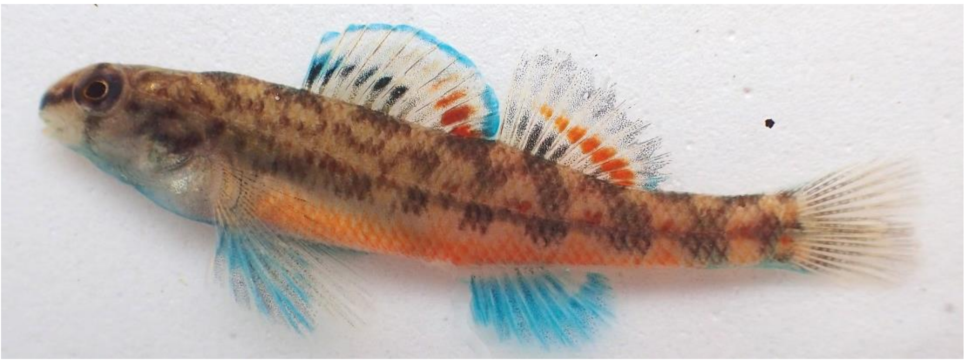

YD_77

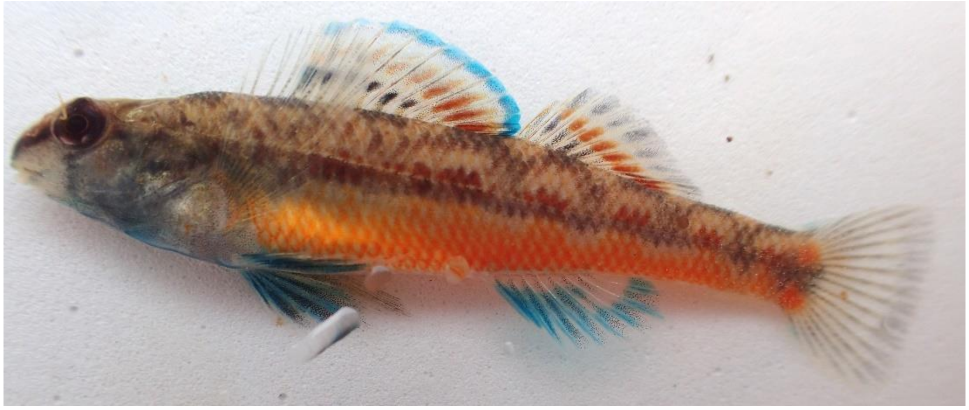

YD_78

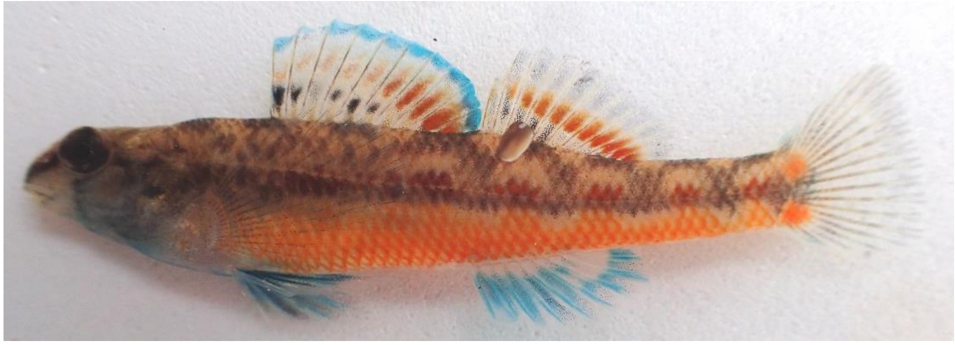

YD_79

Key for location (drainage) data for each photograph; Y.R. = Yocona River drainage and L.T.R. = Little Tallahatchie River drainage

Individual	Drainage
YD_1	Y.R.
YD_2	L.T.R.
YD_3	L.T.R.
YD_4	Y.R.
YD_5	L.T.R.
YD_6	Y.R.
YD_7	Y.R.
YD_8	L.T.R.
YD_9	Y.R.
YD_10	Y.R.
YD_11	L.T.R.
YD_12	L.T.R.
YD_13	L.T.R.
YD_14	Y.R.
YD_15	L.T.R.
YD_16	L.T.R.
YD_17	Y.R.
YD_18	L.T.R.
YD_19	Y.R.
YD_20	Y.R.
YD_21	L.T.R.
YD_22	L.T.R.
YD_23	L.T.R.
YD_24	L.T.R.
YD_25	L.T.R.
YD_26	Y.R.

YD_27	L.T.R.
YD_28	Y.R.
YD_29	Y.R.
YD_30	L.T.R.
YD_31	L.T.R.
YD_32	Y.R.
YD_33	L.T.R.
YD_34	Y.R.
YD_35	Y.R.
YD_36	L.T.R.
YD_37	L.T.R.
YD_38	L.T.R.
YD_39	L.T.R.
YD_40	L.T.R.
YD_41	L.T.R.
YD_42	Y.R.
YD_43	Y.R.
YD_44	L.T.R.
YD_45	Y.R.
YD_46	Y.R.
YD_47	Y.R.
YD_48	L.T.R.
YD_49	Y.R.
YD_50	L.T.R.
YD_51	L.T.R.
YD_52	Y.R.
YD_53	L.T.R.
YD_54	Y.R.
YD_55	L.T.R.
YD_56	L.T.R.
YD_57	Y.R.
YD_58	L.T.R.
YD_59	L.T.R.
YD_60	Y.R.
YD_61	L.T.R.
YD_62	L.T.R.
YD_63	Y.R.
YD_64	L.T.R.
YD_65	Y.R.
YD_66	L.T.R.
YD_67	L.T.R.
YD_68	L.T.R.
YD_69	Y.R.

YD_70	Y.R.
YD_71	L.T.R.
YD_72	L.T.R.
YD_73	L.T.R.
YD_74	L.T.R.
YD_75	Y.R.
YD_76	Y.R.
YD_77	Y.R.
YD_78	L.T.R.
YD_79	L.T.R.

Literature Cited

Adams, S.B. and M.L. Warren, Jr. 2005. Recolonization by warmwater fishes and crayfishes after severe drought in Upper Coastal Plain Hill streams. Transactions of the American Fisheries Society 134:1173-1192.

Boschung, H.T. and R.L. Mayden. 2004. Fishes of Alabama. Smithsonian Books, Washington, D.C. 736 pp.

Brogdon, S.M., C.R. Tabit and L.G. Kral. 2003. Population structure of the Tallapoosa Darter (*Etheostoma tallapoosae*). Southeastern Naturalist. 2:487-498.

Gabel, J.M. 2007. Microsatellite analysis of genetic population structure and gene flow in populations of the federally threatened Cherokee Darter, *Etheostoma scotti*. Unpubl. PhD Dissertation. Duquesne University, Pittsburg, PA. 294 pp.

Hubbell and Banford. 2019. Microhabitat use, spawning behavior, and spawning substrate use of the Tallapoosa Darter (*Etheostoma tallapoosae*). American Midland Naturalist 182:216–227.

Johnston, C.E. and W.R. Haag. 1996. Life history of the Yazoo Darter (Percidae: *Etheostoma raneyi),* a species endemic to north-central Mississippi. Tulane Studies in Zoology and Botany 30:47-60.

Kozal, L.C., J.W. Simmons, J.M. Mollish, D.J. MacGuigan, E. Benavides, B.P. Keck and T.J. Near. 2017. Phylogenetic and morphological diversity of the *Etheostoma zonistium* species complex with the description of a new species endemic to the Cumberland Plateau of Alabama. Bulletin of the Peabody Museum of Natural History 58:263-286.

Ross, S.T. 2001. Inland Fishes of Mississippi. University Press of Mississippi, Jackson, MS. 624 pp.

Sterling, K.A., M.L. Warren, Jr. and L.G. Henderson. 2013. Conservation assessment of the Yazoo Darter (*Etheostoma raneyi*). Southeastern Naturalist 12:816–184.

Storey, C.M. 2003. Genetic population structure and life history aspects of the federally threatened Cherokee Darter, *Etheostoma scotti*. Unpublished thesis, the University of Georgia, Athens. 98 pp.

Suttkus, R.D., R.M. Bailey, H.L. Bart, Jr. 1994. Three new species of *Etheostoma*, subgenus *Ulocentra*, from the Gulf Coastal Plain of southeastern United States. Tulane Studies in Zoology and Botany 29:97-126.
